# Striatal Activity is Associated with Deficits of Cognitive Control and Aberrant Salience for Patients with Schizophrenia

**DOI:** 10.3389/fnhum.2015.00687

**Published:** 2016-02-03

**Authors:** Alan E. Ceaser, Deanna M. Barch

**Affiliations:** ^1^Cognitive Control and Psychopathology Laboratory, Department of Psychology, Washington University in St. LouisSt. Louis, MO, USA; ^2^Department of Psychiatry, Washington University in St. LouisSt. Louis, MO, USA; ^3^Department of Radiology, Washington University in St. LouisSt. Louis, MO, USA

**Keywords:** fMRI, basal ganglia, cognitive control, interference control, prefrontal cortex, striatum, psychosis, aberrant salience

## Abstract

A recent meta-analysis has shown that a large dopamine abnormality exists in the striatum when comparing patients with schizophrenia and controls, and this abnormality is thought to contribute to aberrant salience assignment (or a misattribution of relevance to irrelevant stimuli). This abnormality may also disrupt striatal contributions to cognitive control processing. We examined the relationship between striatal involvement in cognition and aberrant salience symptoms using a task of cognitive control that involves updating, interference control, and simple maintenance. The current study included a sample of 22 patients with schizophrenia and 20 healthy controls and used a slow event-related fMRI design. We predicted that (1) aberrant salience symptoms would be greater for patient's, (2) patients would demonstrate increased errors during interference control trials, given that patients may be inappropriately assigning salience to distracters, and (3) striatal activity during those errors would be correlated with aberrant salience symptoms. We found a trend toward a significant difference between patients and controls on aberrant salience symptoms, and a significant difference between groups on select task conditions. During interference control trials, patients were more likely to inappropriately encode distracters. For patients, both prefrontal and striatal activity was significantly greater when patients inappropriately identified the distracter as correct compared to activity during distracter rejection. During updating, patient prefrontal and striatal activity was significantly lower for incorrect than correct updating trials. Finally, as predicted, for patients the increase of activity during incorrect distracter trials was positively correlated with aberrant salience symptoms, but only for the striatal region. These relationships may have implications for treatments that improve cognitive function and reduce symptom expression.

## Introduction

Schizophrenia is a complex clinical disorder that has a devastating impact on those who suffer from it. In addition to symptoms like hallucinations and delusions, individuals with schizophrenia experience deficits of cognition in the domains of attention and cognitive control, for example, that further complicates the lives and treatment outcomes for individuals with this disorder. The most recent version of the dopamine hypothesis (Howes et al., 2009) suggested that the locus of striatal dopamine dysregulation lies with presynaptic dopaminergic control, which impacts baseline synaptic dopamine levels, dopamine release, and dopamine synthesis capacity. This hypothesis is supported by a meta-analysis of PET studies examining various possible dopamine abnormalities, which found consistent evidence across studies of elevated presynaptic dopamine of patients (Cohen *d* = 0.79; Howes et al., 2012). This pattern was true even when excluding studies with patients receiving antipsychotic medication. This dopamine abnormality is present across all subdivisions of the striatum, but may be most pronounced in the associative striatum (Kegeles et al., 2010). We were interested in examining the relationship between deficits of cognitive control and striatal dysfunction for patients with schizophrenia, as well as determining whether there is a relationship between cognitive control deficits, striatal dysregulation, and symptoms of psychosis.

### Aberrant salience

The relationship between the dopamine abnormality discussed above and psychosis is discussed in the context of the motivational incentive salience literature. The motivational salience hypothesis suggests that dopamine mediates the conversion of a neutral external stimulus into one that is attractive or aversive (Berridge and Robinson, 1998). They distinguish incentive salience from “bottom-up” salience processing, where inherent stimulus properties result in attention capture (e.g., a flashing light or alarm sound), instead stating that motivational salience is a learned, but dynamic, context driven top-down response that transforms neutral stimuli into attractive, desired, relevant incentives that drive behavior. It is influenced by dopamine neurotransmission, but also engages cortical regions as well as structures like the amygdala and nucleus accumbens that interface motivational salience with attention, learning, and cognition. Under normal circumstances, dopamine mediates the acquisition of motivational salience assignment in response to a stimulus based on a person's experience or preference, but it does *not* create this process *independently* of stimulus. In psychosis, however, the revised dopamine hypothesis suggests that dysregulated dopamine transmission leads to a *stimulus-independent* release of dopamine, which then leads to aberrant salience assignment to external stimuli as well as internal representations (Kapur, 2003). As such, this hypothesis proposes that dysregulated dopamine release contributes directly to the formation of delusional symptoms via inappropriate attribution of salience to “neutral” events in the environment. The psychotic experience progressively evolves as aberrant salience brings about a heightened “awareness,” where previously irrelevant stimuli in the environment become relevant (e.g., a television advertisement features the same brand of shoes an individual with psychosis owns). Subsequently, these individuals may feel driven to act on and/or explain the newly relevant phenomenon; at which point a top-down cognitive explanation is imposed (e.g., the television is communicating information to them specifically). Over time, a delusional framework is created to make sense of these experiences. Hallucinations may evolve in a similar way, but with the initial aberrant salient assignment happening to internal representations—internal thoughts, guilt, etc., (from Kapur, 2003). While these experiences occur in the mental background of most healthy individuals, aberrant salience brings them to the foreground for individuals with psychotic symptoms. Thus, this perturbation of salience assignment may lead to the inappropriate assignment of relevance to irrelevant stimuli and symptoms of psychosis. Further, aberrant salience may be related to what could be considered a core component of cognitive control: interference control, or the ability to ignore or block out task irrelevant information. In other words, abnormal salience assignment may be related to difficulties with cognitive control functions that normally serve to distinguish between relevant and irrelevant information.

### Striatal contributions to interference control and cognitive control

Frank et al. (2001) proposed a model of interference control and information gating during cognitive control. This model suggests that cognitive control demands, like rapid updating, maintenance, interference control, and gating of information into working memory, are executed by an interaction between the basal ganglia and the frontal cortex (also see Gruber et al., 2006). The mechanisms that allow this gating to occur are considered an evolutionary extension of the mechanisms involved in the motor control: the direct and indirect pathways (Smith et al., 1998; Hazy et al., 2007). According to this framework, phasic dopamine release in response to task relevant information would result in increases of activity in both the striatum and the frontal cortex, given that both are responding to task demands, but during the presentation of task irrelevant distracters one would not expect to see increased activity in the striatum and transient activity in the prefrontal cortex (without input from the striatum the prefrontal cortex does not fully process the distracter stimuli). For patients with psychotic symptoms, elevated striatal dopamine increases the likelihood that striatal neurons sensitive to phasic dopamine release respond to stimuli, regardless of its relevance, thereby increasing the likelihood that the information gate would open to allow processing of distracter stimuli.

### Current study

The current study sought to further examine the activation in the cortex and the basal ganglia during select cognitive control processes, and to determine what relationship this activity has with symptoms of psychosis, specifically aberrant salience. We used a task designed to isolate cognitive control events in time (i.e., initial encoding, updating, and probe response) and to examine cognitive control demands including updating, interference control, and maintenance. Previously, we found dissociation between subcortical and cortical regions of the brain during updating and interference control, such that both cortical and subcortical regions displayed robust updating activity during updating, but only cortical regions demonstrated increased interference control activity when compared to a “do nothing” condition (Ceaser et al. in prep). The striatal regions that showed effects of interest were almost entirely left lateral, and with the exception of one region in the caudate were all dorsal caudate or dorsal caudal putamen, regions consistent with the associative striatum. These findings suggest while both cortical and subcortical regions are involved in updating processing, the patterns of activity in frontal cortex and striatum are dissociable, and are generally consistent with the pattern of effects one would predict from computational models of cognitive control.

While there is consistent evidence suggesting that striatal dysfunction is associated with psychosis and some evidence suggesting the striatum may play an important role during information gating during cognitive control (Roth et al., 2006; Collette et al., 2007; Murty et al., 2011), few studies if any have found a relationship between these findings. In this study we tested whether patients demonstrate disrupted striatal activity compared with controls during cognitive control performance using a task designed to isolate different components of cognitive control (e.g., updating, maintenance, and interference control). Of these components, we were particularly interested in interference control as striatal dopamine dysregulation and resulting aberrant salience for patients may impair their ability to ignore distracters during these trials. We also examined whether these disruptions in brain activity were associated with particular aspects of behavioral performance as well as aberrant salience symptoms. We predicted that patients would demonstrate poorer behavioral performance on trails involving distracter resistance but would demonstrate preserved performance during trials involving less complex forms of cognition (e.g., maintenance). We predicted that both patients and controls would show increased prefrontal and dorsal striatal activity during incorrect direct distracter. However, we predicted that increased striatal activity, and not prefrontal activity, would be associated with increased symptom severity of delusions, hallucinations, and the index of aberrant salience. We predicted that striatal activity for both patients and controls would demonstrate this relationship, but that it would be stronger for patients. Finally, we predicted that if symptom expression as associated with inappropriate updating specifically we would see a stronger relationship between striatal activity and symptom expression than prefrontal activity and symptom expression.

## Materials and methods

Participants were recruited through the Conte Center for the Neuroscience of Mental Disorders (CCNMD) at Washington University in St. Louis. The Washington University in St. Louis institution review board for human participants approved this study and written consent was obtained from all participants. We recruited 56 participants (30 individuals with schizophrenia and 26 healthy controls). Of those participants, four were excluded because of excessive head movement while in the scanner, nine were excluded for not completing both phases of the study, and one healthy control was excluded because of aberrant behavioral performance (determined by mahalnobis distance, described below). This left 22 participants in the patient group and 20 healthy controls. Patients were diagnosed using the Structured Clinical Interview for DSM-IV (SCID-IV; First et al., 2002), conducted by a master's-level clinician, who completed SCID-IV training and participated in regular diagnostic training sessions as part of the CCNMD. Controls were given a Mini-International Neuropsychiatric Interview (MINI; Sheehan et al., 1998) to determine history of mental illness. Exclusion criteria for controls included a lifetime history of any psychiatric disorder and having a first-degree relative with a psychotic disorder. Participants in either group were excluded if they met criteria for substance abuse or dependence within the past 6 months, had a clinically unstable or severe medical disorder, head trauma with loss of consciousness, or met DSM-IV criteria for mental retardation. Patients were stable on antipsychotic medication doses for at least 2 weeks before participating in the study.

All participants were given the Vocabulary and Matrix reasoning subtests from the Wechsler Adult Intelligence Scale—Third Edition (WAIS-III; Wechsler, 1997) to assess both verbal and non-verbal intelligence. Socioeconomic status and parental education was assessed by asking participants open-ended questions for each parent about what the parent currently does and what they did for a living most of their life. The answers were classified using a scale similar to the British Registrar General's social classification of occupations where occupations range from 0 (low occupational status) to 45 (high occupational status). We focused on parental socioeconomic status and parental education as they may be a more appropriate way to assess developmental exposure to educational opportunities that could influence cognitive function (Resnick, 1992).

### Clinical rating scales

Clinical symptoms of patients were assessed using the Scale for the Assessment of Negative Symptoms (SANS; Andreasen, 1983) and the Scale for the Assessment of Positive Symptoms (SAPS; Andreasen, 1984). These assessments were conducted by a master's-level clinician. All participants also completed the Chapman Psychosis Proneness Scales (Chapman et al., 1980), which included the Perceptual Aberration Scale, the Magical Ideation Scale, the Physical Anhedonia Scale, and the Social Anhedonia Scale.

We assessed aberrant salience specifically using the Aberrant Salience Inventory (ASI; Cicero et al., 2010). It consists of 29 items created to capture the phenomenological descriptions of the initial experience of psychosis in the literature (Kapur, 2003; Parnas et al., 2003). Cicero et al. (2010) found that the ASI was strongly, positively correlated with scales assessing psychotic-like experiences, including magical ideation and perceptual aberration, and other scales measuring psychosis-proneness. The ASI was also found to be positively correlated with social anhedonia, but the correlation was weaker than the correlation between ASI and other scales assessing psychoisis-proneness. The weaker relationship between ASI and anhedonia was predicted given previous work demonstrating a weaker relationship between psychosis-proneness and social anhedonia (Kwapil, 1998). Further, the ASI was found to be elevated in healthy individuals with elevated psychosis proneness as well as participants with a history of psychosis, even when comparing them with a psychiatric comparison group (Cicero et al., 2010). The utility of the ASI, compared to other scales measuring psychosis proneness, is in its specificity. While other scales, including magical ideation and perceptual aberration (CHAPMAN), the Structured Interview for Prodromal Syndromes (SIPS; Kwapil, 1998), and the Schizotypal Personality Questionnnaire (SPQ; Raine, 1991), contain items that are similar to aberrant salience there are other items that may tap into constructs that are related, but peripheral to the core construct of aberrant salience.

### Task design and stimuli

**Figure 1:**
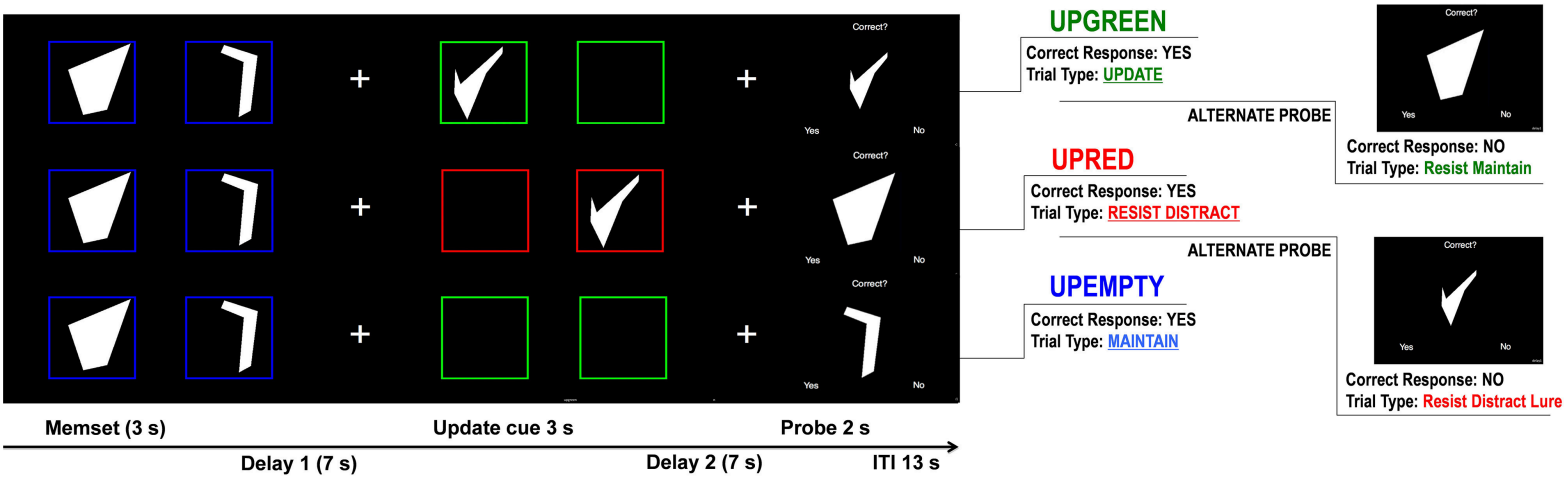
**Controlled Update Task Design**. Trials representing the 3 update cue events (Upgreen, Upred, and Upempty). For each trial participants are first shown two shapes, the first one for 1.5 s and then a second for 1.5 s. They are instructed to remember these shapes in the order that they were presented. After a 7 s delay (Delay 1), 1 of 3 update cue conditions occurs. During the Upgreen condition participants are shown either 1 or 2 new shapes (one after another) framed in green and are tasked with replacing 1 or both of the corresponding memory set items. During the Upred condition participants are shown 1 or 2 new shapes framed in red and are instructed to ignore these shapes and to continue remembering the items from the memory set. During the Upempty condition participants are shown empty boxes that are either red or green. They are told that if no new shape is presented during the update cue they are to simply continue remembering the items from the memory set. Each box during the update cue is presented for 1.5 s. A second delay (Delay 2) follows the update cue, after which the probe is presented for 2 s. During the probe participants are presented a shape and asked if it matches one of the shapes that they are currently remembering. They respond by pressing a “yes” button or a “no” button. Probe types vary for each update cue condition. During Upgreen, for example, participants can be probed with a shape presented during the update cue that they should have remembered (Update trial type), to which they should respond “yes.” Or, during an Upred trial participants can be probed with an item presented during the update condition that they should have ignored (Resist Distracter Lure trial type), to which they should respond “no.”

While in the scanner subjects completed a modified Sternberg-type delayed match-to-sample working memory task. The task contains a two-item working memory load consisting of two complex geometric shapes (Attneave and Arnoult, 1956) that were generated using a Matlab algorithm (Collin and McMullen, 2002). These stimuli were chosen because they may be more difficult to encode than words or numbers. Using shapes may limit encoding strategies used by subjects, which may make difficulty for both patient and control groups more comparable as participants are less likely to spontaneously engage in such verbal maintenance strategies. The shapes were white on a black background and each trial of the task consisted of three distinct, temporally isolated, events: memory set presentation, update cue presentation, and probe presentation (see Figure 1).

### Task design: Memory set

During the memory set participants were presented with two shapes, one after another, framed in a blue box. The shapes were presented for 1.5 s each. Participants were asked to memorize these shapes in the order that they were presented. After the second shape participants saw a fixation cross in the center of the screen that was presented for 7 s. Participants were instructed to focus on the cross while maintaining the previously presented items (Delay 1 in Figure 1).

### Task design: Update cue

After the first delay participants were presented with the update cue items: 2 green or red empty boxes presented one after another for approximately 1.5 s each. If the boxes were green (an Upgreen trial, part A in Figure 1) and a shape appeared in one or both of the boxes, participants were asked to replace the original shape that appeared in that position (the first or second shape that was framed in blue during memory set presentation). During an Upgreen trial participants made either a partial (one shape, either in the first or second position) or a whole update of the original shapes presented during the memory set. If the boxes were red (a Upred trial; part B in Figure 1) participants were asked to ignore any new shapes that were presented and continue remembering the original shapes framed in blue. If, during the update cue, both boxes were empty (an Upempty trail; part C in Figure 1) participants were not required to do anything but maintain the original shapes of the memory set that were framed in blue. Boxes during Upempty trials could be either red or green. Because no new shape was presented participants were instructed that the color of the boxes was irrelevant.

### Task design: Probe and probe types

At the presentation of the probe, participants were presented with one shape, the word “Correct?” appeared at the top of the screen, and at the bottom the word “Yes” appeared on the right and the word “No” appeared on the left. Participants were asked to make a button press if the shape that was presented matched one of the two shapes that they were currently remembering. There were a total of 120 trials used in the task (52 Upgreen, 48 Upred, and 20 Upempty; Supplementary TableA). A number of differing probe types were used in the task to capture a variety of errors that an individual could make during task performance. For example, during Upgreen trials the participant was probed with probed with the item they should have updated. This trial was called an “Update” trial. A correct response indicates that an appropriate update was made and that the new information was encoded into memory. There were a total of 20 Update trials. Another Upgreen probe we used was called a “Resist Maintenance” trial, and participants were probed with the shape in the original memory set that should have been replaced by the new item during the update cue. A correct rejection of this shape indicates that the subject rejected this item as one of the two correct shapes, but a response of “yes” to this items suggests that the participant incorrectly maintained this item in the target set. This type of trial is called a Resist Maintenance trial because participants must resist maintaining this shape when they were being asked to replace it during the update cue. There were a total of 20 Resist Maintenance trials. For trials where the participant was asked to ignore information (Upred), we probed participants with one shape from the original memory set. A correct response on this trial type suggests that the participant was able to maintain information even when presented with task relevant distracters during the update cue period. These trials are called “Resist Distracter” trials and there were 20 of these trials during the task. Another probe type that was used during Upred trials involved participants being probed with a shape that they were asked to ignore during the update cue. These trials are called “Resist Distracter Lure” trials and there were also 20 trials of this type in the task. A correct response during this trial type indicates that a participant correctly rejected a shape that did not match one of the to-be-remembered shapes. An incorrect response on this trial type suggests that the participant inappropriately encoded this shape into memory. Dysregulated salience assignment may lead to increased errors on this trial type, as task information designated as irrelevant may be inappropriately assigned some relevance. Finally, for Upempty trials participants were probed with an item from the original memory set. There were 14 of these trials and they are called “Maintenance” trials. A correct response indicates that the participant correctly maintained this information over the course of the trial. In addition to the probes used in the above mentioned trials participants were probed with shapes that were not presented previously. These trials are called “Novel Probe” trials (26 trials of this type) and were included to ensure that participants could reject probes that were obviously incorrect, and thus these trials gave us a measure of how well participants understood the task's instructions.

### fMRI acquisition

Structural and blood-oxygen-level-dependent (BOLD) data was acquired with a 3T Tim TRIO scanner (Siemens, Malvern, Pennsylvania) at Washington University. Stimuli were projected behind the scanner, visible through a mirror above the eyes. Subjects completed 120 task trials over the course of 10 bold runs. The various trial types were, to the extent possible, evenly interspersed within the 10 runs. Twelve trials occurred in each run. Each trial lasted 35 s (Figure 1). Functional images were acquired using a gradient echo echo-planar sequence maximally sensitive to BOLD contrast (T2^*^; repetition time [TR] = 2000 ms, echo time [TE] = 27 ms, field of view [FOV] = 256 mm, flip = 90°, voxel size = 4 mm^3^). Subjects completed a 7.38-min BOLD run comprised of 210 volumes containing oblique axial images (35 slices per volume) which was acquired parallel to the anterior-posterior commissure. Structural images were acquired using a sagittal MP-RAGE 3D T1-weighted sequence (TR = 2400 ms, TE = 3.16 ms, flip = 8°; voxel size = 1 × 1 × 1 mm).

Preprocessing included: (1) Slice-time correction, (2) Elimination of odd/even slice intensity differences given interpolated acquisition, (3) Rigid body motion correction (Ojemann et al., 1997), (4) Intensity normalization to a whole-brain mode value of 1000 without bias or gain field correction, (5) Registration using a 12-parameter affine transform of the structural image to a template image in the Talairach coordinate system (Talairach and Tournoux, 1988), (6) Co- registration of fMRI volumes to the structural image with 3 mm re-sampling (Ojemann et al., 1997; Buckner et al., 2004), and (7) Smoothing using a 6 mm full-width at half-maximum (FWHM) Gaussian kernel.

### Quality control

We compared the two groups on movement indices and SNR to determine whether there were group differences in these factors that may be influencing group differences in fMRI results. If there were, we confirmed the results of analyses below using subsets of patients and controls matched for movement and SNR. We also used techniques discussed by Siegel et al. (2013) to remove from GLM estimation volumes in which head motion exceeded a threshold (0.5 mm of frame displacement). Participants who lost greater than 40% of the total number of frames, or more than 84 of the 210 frames, were excluded from further analysis.

## Data analysis

### Demographics and behavioral data

We conducted a Mahalanobis distance analysis on the task variables to identify multivariate outlier values, or cases where an individual is responding differently compared to other participants across multiple dimensions. Mahalanobis distance was calculated separately for patients and control for accuracy (including trial types Maintenance, Resist Distracter, Resist Distracter Lure, Update, and Resist Maintenance trials). The probability of distance values were computed separately for patient and control groups. Mahalanobis distance values were assessed using [χ^2^(5, *N* = 43) = 11.07, *p* < 0.05], where values with a probability of less than 0.05 were determined to be outliers and were removed from further analyses.

Chi-squared tests were used for categorical variables (gender, ethnicity) to determine if these distributions differed between groups. We conducted *t*-tests on age, education level, parental education, symptom scores, and measures of IQ (standard scores of verbal and non-verbal IQ) to determine if these variables differed between diagnostic groups. Independent Mann-Whitney U tests were done for variables that failed to demonstrate variance equality.

With regard to task data, because we were primarily interested the Update and Resist Distracter Lure trials, we conduced at repeated measures ANOVA, with trial type (two levels; Update and Resist Distracter Lure trials) as the within subject factor and diagnosis (two levels; patients and controls) as the between subject factor. We were particularly interested in these trials because behavioral accuracy is critically dependent on intact gating functioning. Planned contrasts were done when appropriate to determine whether patient performance significantly differed from controls. A secondary repeated measures ANOVA was done that included the remaining task trials, with trial type (six levels; Maintenance, Resist Distracter, Resist Maintenance, and the three novel probe trial types) as the within subject factor and diagnosis (two levels; patients and controls) as the between subject factor.

### fMRI data analysis

We predicted with when patients are tasked with ignoring distractors they would demonstrate *increased* activity during incorrect trials relative to correct trials. To test these predictions, we examined activity during the update cue phase for specific probe types used in the task (i.e., Update and Resist Distracter Lure) as a function of trial accuracy, as opposed to examining the broad update cue conditions (i.e., Upgreen, Upred, and Upempty) as a function of accuracy. We did attempt to replicate our previous findings (Ceaser et al., in prep) using this new sample of controls, and we conducted an analysis examining update cue conditions. These results can be found in Supplementary Section D (Data Sheet).

The benefit of examining individual trial types is that we can test predictions about specific types of errors. For the Update trial type, an error indicates that the participant rejected an item that was presented during a green update cue, suggested that this item was not appropriately incorporated into the participant's active memory set. Looking at this specific type of error is more informative than looking at any type of error a participant could make during the Upgreen condition, as these could involve failing to identify an item that should have been updated, or incorrectly identifying the to-be-replaced item or incorrectly identifying the novel probe as correct. In the case of Resist Distracter Lure trials, a correct response indicates that participants correctly rejected a response probe that was previously presented as a distracter. If a participant makes an error on this trial type, it indicates that the participant incorrectly accepted the response probe that was previously presented as a distracter, suggesting that they made an inappropriate update. Errors made for the Upred condition, on the other hand, could be the result of an incorrect acceptance of a distracter, but it could also result from participants forgetting the original memory set item, or incorrectly identifying a novel probe as correct.

We were also specifically interested in whether regions in the prefrontal cortex and basal ganglia, specifically the dorsal striatum, demonstrated condition effects. Thus, we used anatomical masks of the basal ganglia (Wang et al., 2008) and the prefrontal cortex (Rajkowska and Goldman-Rakic, 1995), and examined voxel-wise analyses of brain activity within these masks. These ROIs were combined into a single mask and we used a small volume type I error correction, implemented via the Analysis of Functional Neuroimages AlphaSim, of *Z*>2.32, *k* = 20 voxels for this combined ROI mask.

We conducted two repeated measures ANOVA (one examining Resist Distracter Lure performance and one examining Update performance), with accuracy diagnosis as the between subject factor (two levels, patients and controls) and both accuracy (two levels, correct and incorrect) and timepoint (15 frames) as within subject factors. For any significant regions, we conducted a second repeated measures ANOVA for each trial type of interest with diagnosis (two levels), accuracy (two levels), and time (five levels; frames 8–12) as factors, to determine whether the effects reflected group differences during the update component of the trial. We focused our analyses on regions that demonstrated either an interaction of diagnosis by accuracy, or the 3-way interaction of diagnosis by time by accuracy.

### Relationship between symptoms and brain activity analysis

The symptom analysis focused on Magical Ideation, Perceptional Aberration, Social Anhedonia, and Physical Anhedonia from the Chapman scales, as well as the total score from the ASI. We first sought to replicate the relationships between ASI and other measures of psychosis proneness and anhedonia observed previously by Cicero et al. (2010), where strong positive correlations between ASI scores, Magical Ideation, and perceptual aberration were found. We included measures of anhedonia (Social and Physical) with the expectation that there would be little or no correlation between measures of anhedonia and measures of psychosis proneness. By including measures of anhedonia we could assess whether the relationships between cognition, brain functioning, and symptoms were specific to individual symptom domain (e.g., psychosis proneness) or if they generalized to multiple symptom domains (e.g., psychosis proneness and anhedonia).

To examine the relationship between brain activity and symptom expression we first restricted our analysis to regions from the Trial Type Accuracy Analysis that demonstrated sensitivity to differences in accuracy during Resist Distracter Lure and Update trials during the time period following the presentation of the update cue (frames 8–12). We then extracted the average magnitude of activity from the five time points of interest for these regions and ran Pearson's correlation analyses between the average of these time points and symptom scores. We predicted that patients would display a significant positive correlation between brain activity, specifically dorsal striatal activity, associated with the update cue during Distracter Resistance Lure trials and ASI, but only when incorrect responses were made to the probe. We did not predict a correlation between brain activity associated with Distracter Resistance Lure trials and ASI when patients made correct responses to the probe, given that ASI and interference control errors are proposed to result from striatal dysregulation and correct trials are not thought to result from such dysregulation. That is, striatal activity during incorrect Distracter Lure trials may represent instances where dysregulation was sufficient enough to produce false alarms, whereas activity during correct trials was not have sufficient to produce false alarms. Further, as noted above, we did predict to find significant correlations between ASI and psychosis proneness scales (i.e., Magical Ideation and Perceptual Aberration). Thus, if dorsal striatal activity is positively associated with aberrant salience, we would also expect to see a positive correlation between striatal activity and psychosis proneness scales for patients, but not a strong correlation between striatal activity and measures of anhedonia.

## Results

### Demographics

Demographic data for each group is shown in Table 1. The groups did not differ in gender [χ^2^(1) = 1.43, *p* = 0.23], parental education [*t*_(36)_ = −1.19, *p* = 0.34] or subject education [*t*_(35)_ = −0.99, *p* = 0.9]. However, we did find a significant difference in age [*t*_(40)_ = 2.49, *p* = 0.02], such that the patient group was slightly older than the control group (see Table 1). We also found a marginal difference in ethnic composition [χ^2^(3) = 8.1, *p* = 0.057, φ = 0.42]. Given these differences in age and ethnicity we used these variables as covariates during all planned follow up analyses that explored effects that interacted with diagnosis from the voxel-wise analyses.

**Table 1:** **Demographics, task data, and symptom scores for patients and controls**.

	**SCZ (*N* = 22)**	**Controls (*N* = 20)**	**Sig**.	**ES (*d)***
**DEMOGRAPHICS**					
Age	40.41 (8.4)	33.65 (9.19)	0.02	0.77
Gender	75% Male	55% Male	0.23	
Race	11 AA, 10 Cau	14 AA, 2 Asian, 2 Cau, 1 Other	0.06	0.42
Subject education (Years)	15.0 (3.51)	15.1 (2.59)	0.9	
Parental education (Years)	13.54 (4.74)	14.7 (3.1)	0.34	
**NEUROPSYCHOLOGICAL TESTING/QUESTIONNAIRES**					
IQ (WAIS III—Vocab)	91.36 (17.33)	100.5 (14.32)	0.07	
IQ (WAIS III—Matrix)	103.86 (14.55)	105.5 (12.24)	0.7	
Aberrant salience inventory (ASI)	13.59 (8.29)	9.05 (6.88)	0.06	0.6
Chapman—Perceptual Aberration^*^	7.59 (9.23)	2.25 (2.48)	0.03	0.79
Chapman—Magical Ideation^*^	11.18 (7.27)	5.10 (4.41)	0.001	1.01
Chapman—Social Anhedonia	19.64 (9.04)	9.35 (6.02)	0.001	1.34
Chapman—Physical Anhedonia^*^	24.41 (12.68)	10.7 (6.37)	0.001	1.37
**EXPERIMENTAL TASK**					
Resist distracter	0.63 (0.23)	0.69 (0.19)	0.34	
Resist distracter lure^*^	0.52 (0.26)	0.74 (0.19)	0.006	0.97
Maintenance^*^	0.67 (0.22)	0.7 (0.16)	0.46	
Update	0.72 (0.17)	0.82 (0.13)	**0.03**	0.66
Resist maintenance^*^	0.49 (0.29)	0.69 (0.16)	0.02	0.85
Resist distracter novel probe	0.60 (0.30)	0.81 (0.25)	0.02	0.76
Maintenance novel probe^*^	0.64 (0.31)	0.81 (0.21)	0.04	0.64
Update novel probe^*^	0.61 (0.32)	0.84 (0.18)	0.02	0.88

*Demographics, cognitive scores, symptom scores, and task data for both patients and controls. P-values of differences between groups are listed under the heading “Sig.” Significant p-values are printed in red text. Effect sizes (Cohen's d) for significant between group differences are listed under the heading “ES (d).” Variables with an asterisk failed tests of equal variances between groups. The p-values for these variables were generated using nonparametric tests*.

### Clinical and cognitive measures

While controls had numerically higher scores on measures of verbal and non-verbal IQ, these differences were not statistically significant (verbal IQ trended toward significance [*t*_(40)_ = −1.85, *p* = 0.07)]. We observed significant differences between the groups on most symptom measures, including all Chapman scales (Magical Ideation, Perceptual Aberration, Social Anhedonia, and Physical Anhedonia; Table 1). Nonparametric tests were used to compare Perceptual Aberration, Physical Anhedonia, and Magical Ideation between groups as these variables failed to demonstrate variance homogeneity. There was a trend of patients and control to differ on the ASI [*t*_(40)_ = 1.92, *p* = 0.06, *d* = 0.6].

**Figure 2:**
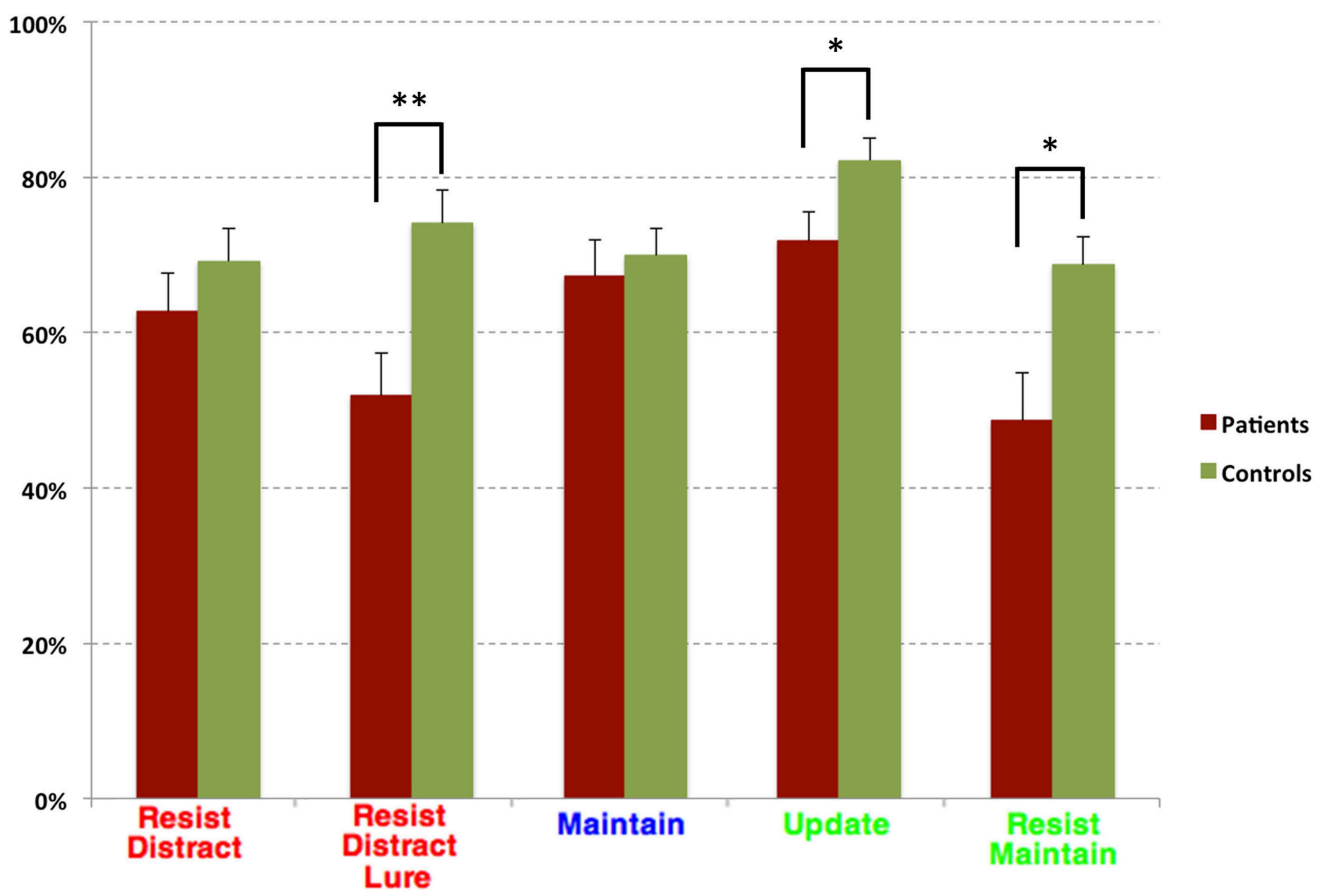
**Task Accuracy for Diagnostic Groups**. Task accuracy for patients (burgundy) and controls (green). Red colored text indicate trail types that were also considered an Upred condition, blue colored text were trial types that were also considered an Upempty condition, and green colored text were also considered an Upgreen condition. While generally patients performed numerically worse than controls on all trial types, these differences were only significant for the Resist Distracter Lure, Update trial types, and Resist Maintenance trials. ^*^p < 0.05 and ^**^p < 0.01.

Task performance for the two diagnostic groups can be seen in Figure 2. Overall, controls performed better than patients. The repeated measures ANOVA of trial type (two levels, Update and Resist Distracter Lure) and diagnosis (two levels, patients and controls) revealed a main effect of diagnosis [*F*_(1, 38)_ = 20.23, *p* < 0.001], but no main effect of trial type [*F*_(1, 38)_ = 1.76, *p* = 0.19], and no interaction of trial type and diagnosis [*F*_(1, 38)_ = 2.7, *p* = 0.11]. *Post-hoc t*-tests (Fisher's LSD) revealed that performance no significant difference between group for the Update trial type [*t*_(41)_ = −1.03, *p* = 0.31] but a significant difference between group for the Resist Distracter Lure trial type [*t*_(41)_ = −2.23, *p* = 0.03] differed between patients and controls, such that patients performed significantly worse on both trial types. Given that we found significant differences of novel probe performance between diagnostic groups (suggesting a global cognitive deficit rather than one specific to distracter resistance, for example) we conducted separate multiple regression analyses to test whether diagnostic group could significantly predict Resist Distracter Lure and Update performance. Diagnostic group trended toward significantly predicting Resist Distracter Lure performance when controlling for novel probe performance [*B* = 0.1, *t*_(41)_ = 1.88, *p* = 0.07]. Diagnostic group did not predict Update accuracy when controlling for Update Novel Probe performance, trend or otherwise [*B* = 0.08, *t*_(41)_ = 1.33, *p* = 0.19]. A repeated measures ANOVA on the remaining trial types revealed a significant main effect of diagnosis [*F*_(1, 38)_ = 14, *p* = 0.001], a trend toward a main effect of trial type [*F*_(2.5, 38)_ = 2.25, *p* = 0.1], and a significant interaction of diagnosis by trial type [*F*_(2.5, 38)_ = 3.11, *p* = 0.04] when using the sphericity correction. Follow up *t*-tests revealed significant differences between diagnostic groups for all trial types, with the exception of Resist Distracter and Maintenance trial types (see Table 1). For all trial types that demonstrated significant differences between diagnostic groups, patients performed worse that controls.

### Imaging trial type accuracy results

**Figure 3:**
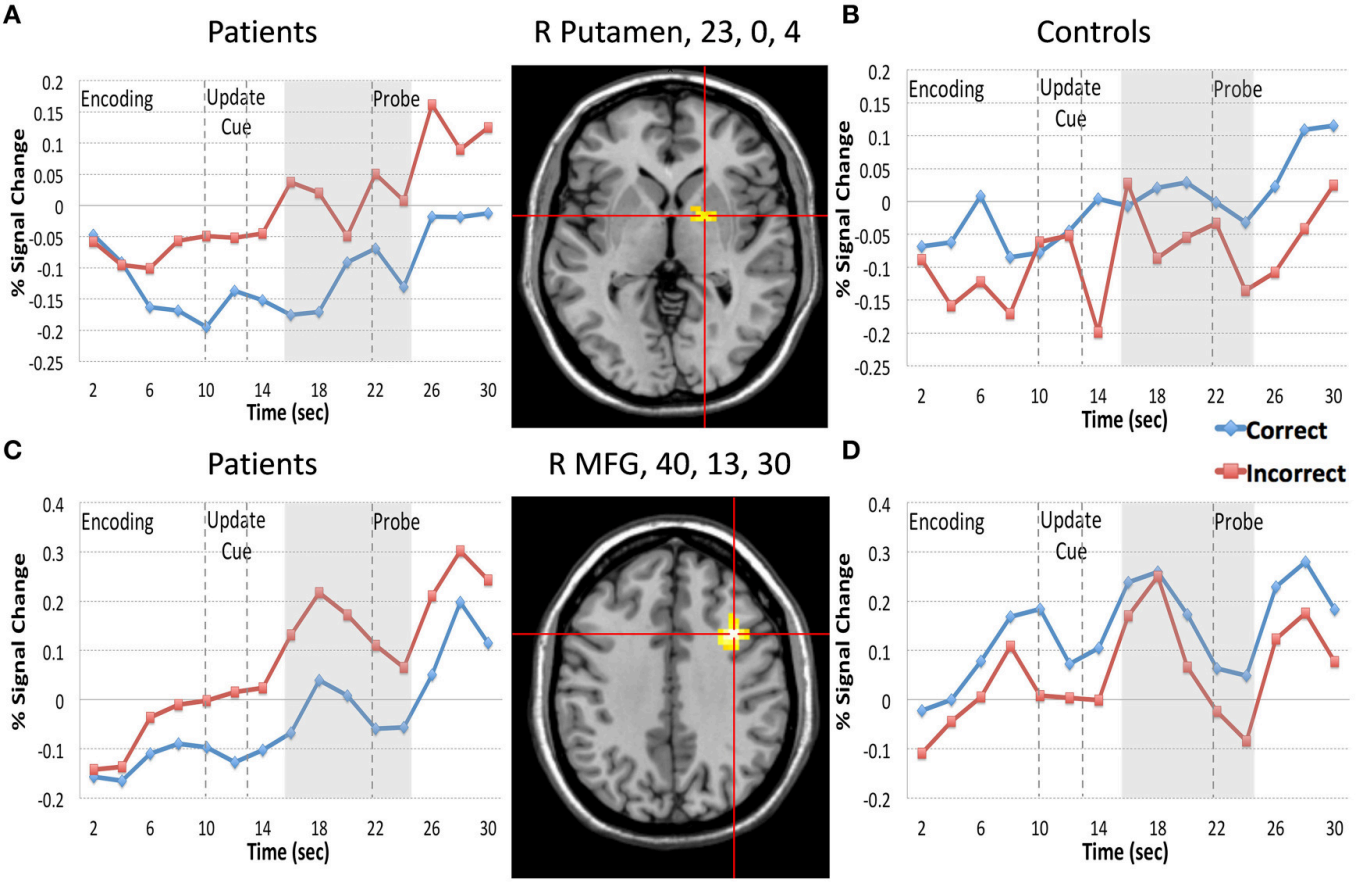
**Regions Demonstrating a Diagnosis by Resist Distracter Lure Trial Type Accuracy interaction**. We plotted the 2 regions (putamen, 23, 0, 4, and MFG, 40, 13, 30) that demonstrated diagnosis by Resist Distracter Lure accuracy following the presentation of the update cue. Red lines in the figure represent incorrect trial activity and blue lines represent correct trial activity. During Resist Distracter Lure participants are probed with an item they were instructed to ignore. If participants respond “no” they are correctly rejecting this item. However, if participants respond “yes” it suggests that they inappropriately updated this item when it was presented. “Memory Set” in the figure denotes the period during which the memory set items are presented. “Update Cue” in the figure and the two arrow lines represent the onset (10 s) and offset (13 s) of the update cue event. The gray box (16–24 s) represents the time frame used in our follow up update cue analyses (corresponding to frames 8–12), which is shifted from the offset of the update cue to account for hemodynamic lag. “Probe” in the figure and the arrow line at the 22 s time point indicate the onset of the probe. For both the putamen (top row of the figure) and MFG (bottom row of the figure) we found that for patients **(A,C)** incorrect trial activity was significantly greater than correct trial activity for frames 8–12 (16–24 s during the trial), consistent with the idea that increases of brain activity in these regions are associated with information updating. For controls **(B,D)** we found the opposite pattern, such that incorrect trial activity was significantly less than correct trial activity for the putamen, and numerically, but not significantly, less than correct trial activity in the MFG.

We first focused on the Resist Distracter Lure trial type (Table 2). Regions that demonstrated relevant effects from our whole brain analysis can be seen in the Supplementary Sections B,C. Two regions demonstrated and interaction of accuracy and diagnosis, including the right lateral putamen (23, 0, 4) and right lateral MFG (40, 13, 30) when examining all 15 frames of the trial. When examining whether these regions demonstrated this effect following the presentation of the update cue (in this case, the presentation of a distracter) we found that both regions still demonstrated a significant interaction of diagnosis and accuracy (Table 2). For patients, activity during incorrect trials (where, at the probe, the identified the distracter presented during the update cue as a correct response) was significantly greater than trials when they correctly rejected the distracter at the probe. This was true for both the putamen (Figure 3A) and the MFG (Figure 3C). Controls, however, did not show this pattern. If anything, for controls *correct trial* activity within a region in the putamen trended toward being significantly *greater* than incorrect trial activity following the update cue (Figure 3B), which is the opposite of the pattern observed for patients in this region. The same was true of controls for activity in the MFG, where correct trial activity was numerically greater than incorrect trial activity (Figure 3D).

**Table 2:** **Regions demonstrating an effect of diagnosis by resist distracter lure trial type accuracy within our anatomical masks**.

***X***	***Y***	***Z***	**Size**	**Hemi**	**Region**	**BA**	**Effect at frames 8–12**			**Correct vs. incorrect**	
							**Analysis**	***F***	***p***	**Patients**	**Controls**
**DIAGNOSIS**											
−25	−18	−1	44	Left	Putamen						
−39	35	2	45	Left	IFG	46					
23	51	6	21	Right	SFG	10					
−33	48	13	22	Left	MFG	10					
−33	7	33	25	Left	Precentral Gyrus	9					
**ACCURACY**											
25	55	3	21	Right	SFG	10					
**ACCURACY X TIME**											
23	−14	7	35	Right	Putamen						
**DIAGNOSIS X ACCURACY**											
23	0	4	44	Right	Putamen		Dx X Acc	10.41	0.003	cor < incor^**^	cor > incor^**^
40	13	30	70	Right	MFG	9	Dx X Acc	14.7	<0.0001	cor < incor^**^	no diff

*Regions from the current data set that demonstrated diagnosis by Resist Update Lure accuracy. They are organized on the left side under headings like “Diagnosis” or “Accuracy” based on whether they demonstrated these effects when examining all 15 time frames of the trial. We only conducted follow up analyses for the update cue period on regions that demonstrated a significant interaction of accuracy and diagnosis. Statistics from the update cue response analysis can be found under the heading “Effect at frames 8–12.” “cor,” correct trials and “incor,” incorrect trials*.

** *p < 0.01. “no diff” signifies no statistically significant difference*.

**Figure 4:**
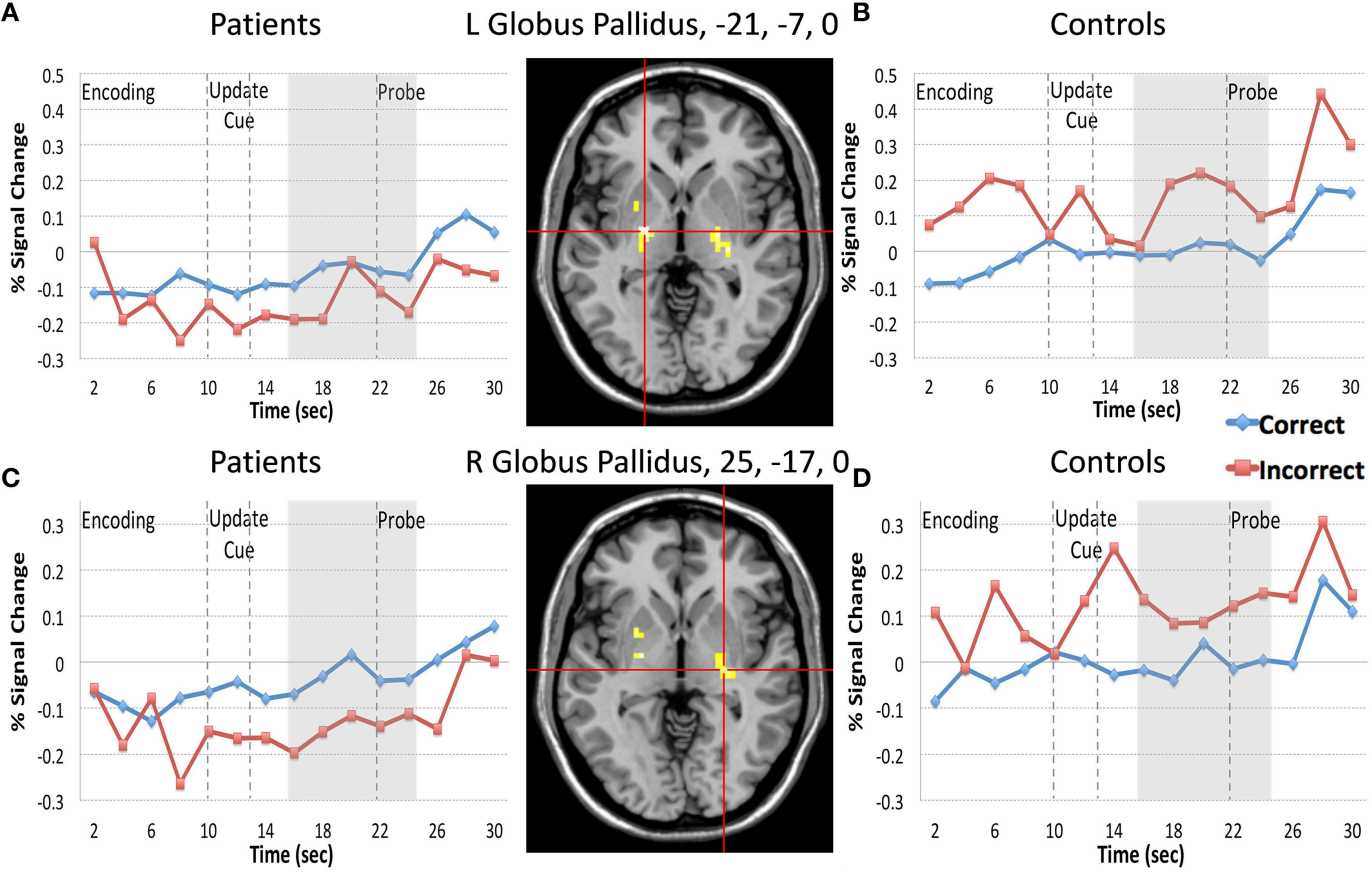
**Regions Demonstrating a Diagnosis by Update Trial Type Accuracy interaction**. We plotted the regions in bilateral globus pallidus that demonstrated diagnosis by Update accuracy following the presentation of the update cue. Red lines in the figure represent incorrect trial activity and blue lines represent correct trial activity. For the Update trial type participants are probed with an item they should have updated during the update cue. A “yes” response indicated they made the appropriate update, and a “no” response suggests that they did not. For both regions of the globus pallidus patients **(A,C)** demonstrated significantly less activity during incorrect trials when compared with correct trials, again consistent with the idea that increases of brain activity in these regions are associated with information updating. However, for controls **(B,D)** we again found the opposite pattern to patients when comparing correct and incorrect trial activity. Controls, on the other hand, demonstrated the opposite pattern of patients, such that activity in the left globus pallidus during incorrect trials following the presentation of the update cue was significantly greater than activity during correct trials. Activity in the right globus pallidus for controls did not significantly differ when comparing correct and incorrect Update activity following the presentation of the update cue.

Next we examined the Update trial type. When examining all 15 time frames there were two regions that demonstrated an interaction of diagnosis and accuracy (bilateral globus pallidus, −21, −7, 0, and 25, −17, 0) and two regions that demonstrated a Three-way interaction of diagnosis by time by accuracy (bilateral IFG, including 40, 43, 1, and −42, 8, 31). When examining whether these regions continued to interact with diagnosis and accuracy in analyses restricted to the frames following the presentation of the update cue (frames 8–12) we found that two regions from bilateral globus pallidus and one region in the IFG demonstrated significant interactions of diagnosis by accuracy (Table 3). For patients, activity in both regions of the globus pallidus significantly differed when comparing correct and incorrect Update activity following the presentation of the update cue, such that correct activity was greater than incorrect activity (Figures 4A,C). For controls, correct and incorrect activity in these regions also significantly differed from one another, however incorrect trial activity in both regions was greater than correct trial activity. The pattern for controls when comparing correct Update trial activity to incorrect Update trial activity was the opposite of the pattern of correct vs. incorrect Update trial activity for patients (Figures 4B,D). This was unexpected.

**Table 3:** **Regions demonstrating an effect of diagnosis by update trial type accuracy within our anatomical masks**.

***X***	***Y***	***Z***	**Size**	**Hemisphere**	**Region**	**BA**	**Effect at frames 8–12**			**Correct vs. Incorrect**	
							**Analysis**	***F***	***p***	**Patients**	**Controls**
**DIAGNOSIS**											
24	−9	4	222	Right	Putamen						
14	3	15	92	Right	Caudate Body						
−22	−8	5	251	Left	Globus Pallidus						
−40	38	7	211	Left	IFG	46					
30	30	−5	40	Right	IFG	47					
23	52	3	23	Right	SFG	10					
39	18	28	133	Right	MFG	9					
−39	8	34	42	Left	Precentral Gyrus	9					
**ACCURACY**											
−36	8	29	34	Left	IFG	9					
44	8	32	38	Right	MFG	9					
**ACCURACY X TIME**											
11	−1	14	27	Right	Caudate Body						
35	34	−5	28	Right	MFG	47					
37	28	29	87	Right	MFG	9					
−44	11	29	64	Left	IFG	9					
**DIAGNOSIS X TIME**											
44	40	−2	49	Right	Sub-Gyral	10					
−40	44	3	180	Left	IFG	10					
42	18	30	91	Right	MFG	9					
**DIAGNOSIS X ACCURACY**											
−21	−7	0	34	Left	Globus Pallidus		Dx X Acc	9.49	0.004	cor > incor^*^	cor < incor^**^
25	−17	0	34	Right	Globus Pallidus		Dx X Acc	13.99	0.001	cor > incor^**^	no diff
**DIAGNOSIS X TIME X ACCURACY**											
40	43	1	41	Right	IFG	10	Dx X Acc	4.53	0.04	no diff	no diff
−42	8	31	24	Left	IFG	9	Dx X Acc	0.01	0.92		

*Regions from the current data set that demonstrated diagnosis by Update accuracy. They are organized on the left side under headings like “Diagnosis” or “Accuracy” based on whether they demonstrated these effects when examining all 15 time frames of the trial. We only conducted follow up analyses for the update cue period on regions that demonstrated a significant interaction of accuracy and diagnosis. Statistics from the update cue response analysis can be found under the heading “Effect at frames 8–12.” “cor,” correct trials and “incor,” incorrect trials*.

* p < 0.05 and

** *p < 0.01. “no diff” signifies no statistically significant difference*.

Another region, right lateral IFG, also demonstrated a significant effect of diagnosis by accuracy following the presentation of the update cue, but when comparing correct and incorrect Update trial activity within diagnostic groups neither group demonstrated a difference (Table 3). We examined the time course of activity for this region to determine where the effect was coming from. While numerically the pattern of activity in this region was the same for patients and controls as what we observed in the globus pallidus (greater correct than incorrect trial activity following the presentation of the update cue for patients and the opposite pattern for controls), the differences did not reach significance. There did appear to be differences earlier during the trial (around the onset of the memory set) for both patients and controls that may have driven the initial interaction of diagnosis by time by accuracy when we examined all 15 time frames.

### Relationship between symptoms and brain activity results

**Figure 5:**
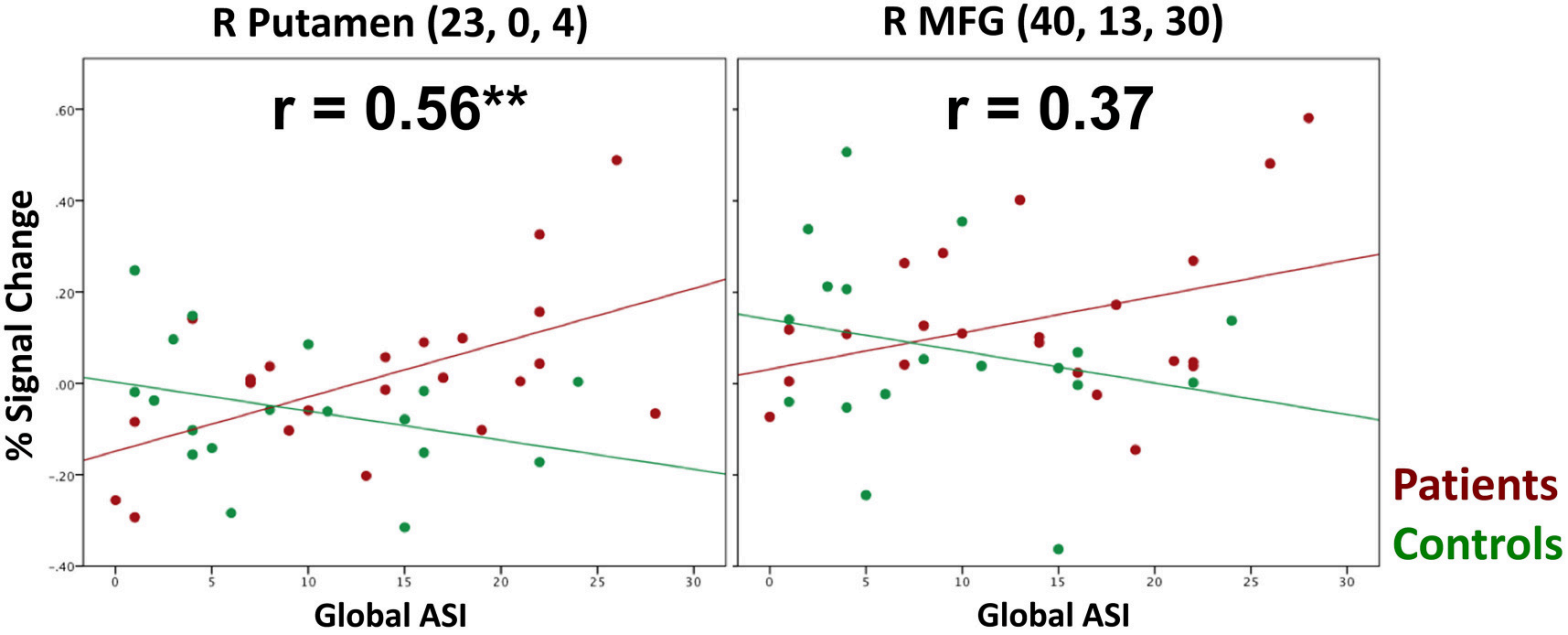
**Scatter Plots Depicting the Relationship Between ASI and Brain activity during Incorrect Resist Distracter Lure Trials**. We observed a significant positive correlation between ASI and brain activity in the right lateral putamen during incorrect Resist Distracter Lure trials for patients but not controls, such that, as predicted, the brain activity for patients who were susceptible to distraction increased as ASI symptoms increased. If anything, putamen activity of controls demonstrated a non-significant correlation in the opposite direction. While we also observed a positive correlation between MFG activity during incorrect Resist Distracter Lure trials and ASI scores for patients, this correlation did not reach significance.

For patients (Table 4, burgundy text), aberrant salience did not significantly correlate with putamen activity in response to the update cue when patients made correct responses. Correct trial putamen activity of patients significantly positively correlated with only one other measure of psychosis proneness—magical ideation. Incorrect trial activity in the putamen of patients following the presentation of the update cue was significantly positively correlated with aberrant salience (Table 4 and Figure 5). Incorrect trial activity in the putamen of patients also demonstrated a significant positive correlation with magical ideation. We tested whether the correlation between ASI and putamen activity for correct vs. incorrect trials differed for patients (Meng et al., 1992), and we found a trend toward significance (*z* = −1.56, *p* = 0.06). Correct activity in the MFG negatively correlated with ASI scores, but this correlation did not reach significance. The direction of the correlation between correct MFG activity and other measures of psychosis proneness, including perceptual aberration and magical ideation, was also negative but these correlations also did not reach significance. We were interested in determining whether the correlation between incorrect trial activity and ASI differed between he putamen and MFG, and found that the correlations did not significantly differ (*z* = 0.8, *p* = 0.21).

**Table 4:**
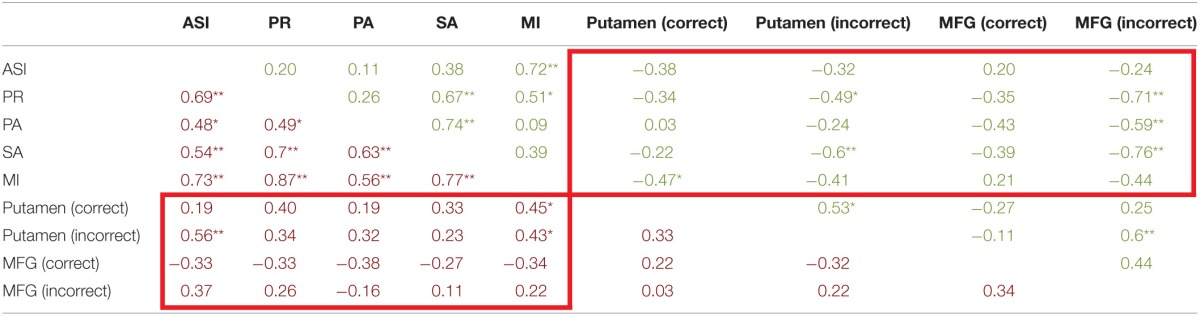
**Correlations between correct and incorrect resist distracter lure trial type brain activity and aberrant salience, psychosis proneness, and anhedonia measures**.

Correlations between Resist Distracter Lure, correct and incorrect, trial brain activity and clinical symptom measures. Patient correlations are printed in burgundy and controls correlations are printed in green. The red boxes indicate correlations of interest. ASI, aberrant salience inventory; PR, perceptual aberration; PA, physical anhedonia; SA, social anhedonia; MI, magical ideation. ^*^p < 0.05 (2-tailed) and ^**^p < 0.01 (2-tailed).

For controls (Table 4, green text), we found no correlation between ASI scores and either correct or incorrect activity for the putamen and MFG (Table 4). Incorrect Resist Distracter Lure activity in the putamen was negatively correlated with perceptual aberration and social anhedonia, but did not significantly correlate with any other measure of psychosis proneness or anhedonia. We found that, when comparing the correlation between putamen activity and social anhedonia, correlations for correct trial and incorrect trial activity significantly differed from one another (*z* = −1.83, *p* = 0.03). However, we did not find that the correlation between correct trial putamen activity and perceptual aberration differed from the correlation between incorrect trial putamen activity and perceptual aberration (*z* = −0.72, *p* = 0.24), nor did the correlation between correct trial putamen activity and magical ideation differ from the correlation between incorrect trial putamen activity and magical ideation (*z* = −0.29, *p* = 0.39). For the MFG, while correct activity did not significantly correlate with any measure of psychosis proneness or anhedonia, incorrect trial activity demonstrated significant negative correlations with perceptual aberration, physical anhedonia, and social anhedonia. The correlation between MFG activity and perceptual aberration significantly differed between correct and incorrect trials (*z* = −1.8, *p* = 0.04) as did the correlation between MFG activity and social anhedonia when comparing correct and incorrect trial activity (*z* = −1.98, *p* = 0.02), but correlations between MFG activity and physical anhedonia did not differ when comparing correct and incorrect trials (*z* = −0.77, *p* = 0.2). Incorrect trial activity in the MFG for controls was not significantly correlated with ASI (Figure 5) or magical ideation, although the direction of the correlations for these variables was also negative. Finally, we examined whether the significant correlation between ASI and incorrect trial putamen activity significantly differed between patients and controls. We found that, indeed, it did (*z* = 2.89, *p* = 0.004).

Next we examined the relationship between aberrant salience and brain activity in regions from the trial type analysis (see above) that demonstrated a significant interaction of diagnosis and Update accuracy. We found that two regions, bilateral globus pallidus (Table 3), during the Update trial demonstrated a significant interaction of diagnosis by accuracy in response to the update cue. For both of these regions patients demonstrated a significant positive correlation between ASI scores and brain activity, but only during correct trials (Table 5). The correlation between ASI and incorrect trial activity significantly differed from the correlation between ASI and correct trial activity for both left (*Z* = 2.26, *p* = 0.01) and right (*Z* = 2.84, *p* = 0.002) lateral globus pallidus. Left lateral globus pallidus activity also significantly correlated with magical ideation in the same direction, but not with perceptual aberration or the anhedonia measures. Right lateral globus pallidus activity, however, demonstrated significant positive correlations with all other measures of psychosis proneness and anhedonia.

**Table 5:**
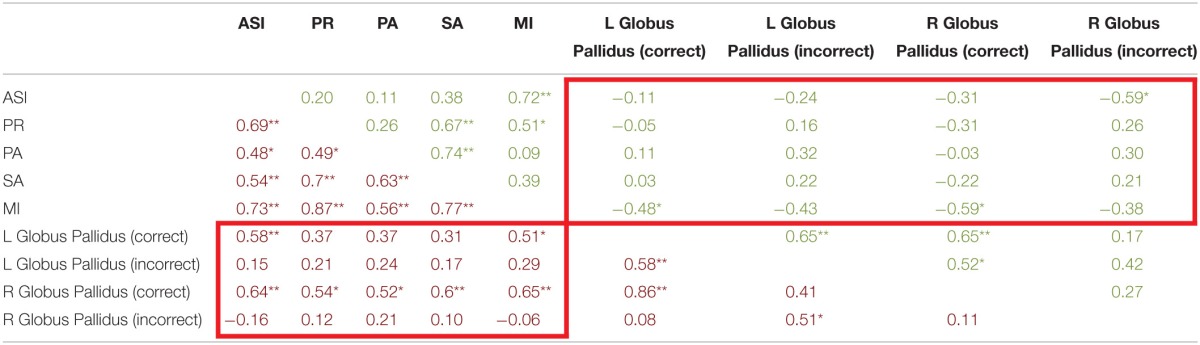
**Correlations between correct and incorrect update trial type brain activity and aberant salience, psychosis proneness, and anhedonia measures**.

Correlations between Update, correct and incorrect, trial brain activity and clinical symptom measures. Patient correlations are printed in burgundy and controls correlations are printed in green. The red boxes indicate correlations of interest. ASI, aberrant salience inventory; PR, perceptual aberration; PA, physical anhedonia; SA, social anhedonia; MI, magical ideation. ^*^p < 0.05 and ^**^p < 0.01.

For controls, we observed a significant negative correlation between ASI and right lateral globus pallidus activity during incorrect Update trials. Activity in this region did not significantly correlate with other measures of psychosis proneness or anhedonia. When we compared the correlation between ASI and right lateral globus pallidus activity during incorrect and correct trials we found that no difference between correlations (*z* = −1.14, *p* = 0.13). While correct trial activity in bilateral globus pallidus did not significantly correlate with ASI, we did observe significant negative correlations with magical ideation (Table 5) bilaterally. Neither correlation between left and right globus pallidus correct trial activity and magical ideation significantly differed from respective incorrect trial correlations between brain activity and magical ideation.

## Discussion

This study sought to test whether individuals with schizophrenia have dysregulated striatal activity when processing cognitive control demands, whether this dysregulation is associated with performance deficits, and whether striatal activity is associated with aberrant salience symptoms. We found evidence to support this hypothesis, as patients performed worse than controls on distracter resistance trials but performance did not differ on maintenance trials. We also found that patients demonstrated increased striatal and prefrontal activity during incorrect distracter resistance trails, when they may have inappropriately updated information. While this finding is consistent with our hypothesis, we did not observe the same pattern for controls. Finally, we predicted that increased striatal activity, and not prefrontal activity during interference control trials, would be associated with increased symptom severity of delusions, hallucinations, and the index of aberrant salience from the ASI. We found evidence to support this hypothesis, as striatal activity of patients during error trials positively correlated with symptom expression. We did not find the same relationship pattern for our control subjects. These findings are discussed further below.

### Trial type accuracy

As predicted we found increased activity for incorrect Resist Distracter Lure trials compared with correct trials within a right DLPFC region and within the right putamen, but for patients not controls. For controls, activity within the DLPFC demonstrated no difference between correct and incorrect trials, and greater correct trial activity than incorrect trial activity within the putamen—the opposite pattern of patients with schizophrenia. Further, we found greater activity during incorrect trials for patients. These findings are consistent with the idea that for patients, activity occurring in response to the update cue, even when instructed to ignore these items, meaningfully relates to later behavioral accuracy at the probe. Further, this later finding fits the prediction one would make based on the computational models of gating (e.g., Frank et al., 2001) described above, where increases of striatal and prefrontal activity are associated with gating information into working memory. Unfortunately, with our current design it is difficult to disentangle the causal contributions prefrontal and striatal regions have on behavioral outcomes, given the relationship between basal ganglia output and prefrontal function described above. For example, it is possible that basal ganglia output precedes prefrontal activation and increases of activity represent information updating whereas prefrontal activity represents storage and maintenance related activity of the updated item. It is also possible that prefrontal activity during distracter presentation may occur first and increases of striatal activity result from downstream effects of cortical activity, perhaps through glutamatergic afferents from the cortex to spiny neurons in the striatum (Rosell and Giménez-Amaya, 1999).

We also examined whether cortical and subcortical brain activity differed between diagnostic groups during correct and incorrect Update trials. Correct responses during Update trials indicate that the participant correctly identified the “to-be-remembered” shape at the probe and incorrect trials indicate that the participant rejected the shape, suggesting they did not update or encode new information as instructed. Again, although correct and incorrect trials are defined by the response made at the probe, we examined activity that was associated with the presentation of the “to-be-remembered” items. Behaviorally, we found that patients and control performance significantly differed for this trial type, although the effect was smaller for the Update (Cohen's *d* = 0.66) vs. the Resist Distracter Lure trial type (Cohen's *d* = 0.97). With regard to brain activity, we found that bilateral globus pallidus activity for patients was significantly *greater* for correct than incorrect trials.

### Involvement of the palladium in cognitive control

In Hazy et al. (2007) model described above, the globus pallidus is associated with the indirect pathway and receives inhibitory input from striatal MSNs. This inhibition activates the globus pallidus, causing disinhibition of substantia nigra pars reticulata (which is tonically inhibited by the globus pallidus), and this disinhibition of the substantia nigra competes with inhibitory input from striatal MSNs associated with the direct pathway. Thus, increases of activation of the globus pallidus should disinhibit the substantia nigra, making it *less* likely that the cortex will be released from thalamic inhibition and *less* likely that an update will occur. Our finding of greater activity on correct vs. incorrect trials in the globus pallidus in patients is not consistent with the predictions of the Hazy model, depending on where in particular our regions of the globus pallidus lie. However, this result might be consistent with the findings of McNab and Klingberg (2008). They found that increases of globus pallidus activity, which preceded the presentation of distracters, was associated with increasing working memory storage. They suggested that the globus pallidus might function as an information filter that increases activity in response to relevant task information and decreases activity in response to irrelevant information. In this context it makes some sense that increases of activity within the globus pallidus are associated with correct trials of information updating as task relevant information is being filtered in and lower activity is associated with errors, but again we only found this pattern of effects for patients and not controls. Of the two globus pallidus regions that demonstrated a significant interaction of diagnosis and accuracy for Update trials, controls only demonstrated a difference between correct and incorrect trial activity within the left lateral globus pallidus, such that correct trial activity was significantly less than incorrect trial activity (Figure 4B)—the opposite pattern that patients displayed.

### Differences between groups in putative information gating engagement

Unexpectedly, we did not find the same pattern of effects for controls that we did for patients. If this cortico-striatal mechanism is indeed a mechanism of gating we should expect to see the same pattern of results regardless of diagnostic status. That is, if controls inappropriately update information it should be reflected in a neural response of the striatum and the prefrontal cortex. The fact that we failed to find differences between correct and incorrect trials for controls or the patterns we did find were the opposite of patients with schizophrenia might suggest that the activity we observed for patients reflects something might be specific to disease state. However, when examining brain activity in regions that showed interactions of accuracy and time for the Resist Distracter Lure and Update trials (e.g., right putamen, Table 2 and right caudate, MFG and left IFG, Table 3), we found that for a region in the putamen that demonstrated an accuracy by time interaction both patients and controls demonstrated numerically greater incorrect Resist Distracter Lure trial activity than correct trial activity. Further, the activity for both patients and controls in regions that demonstrated an effect of Update accuracy by time did not appear to differ when comparing correct and incorrect trials to one another. Thus, we found some evidence that to suggest that the patterns of patient and control activity during correct and incorrect Resist Distracter Lure and Update trials is comparable, but further work is need to determine if the counterintuitive finding that patients and controls demonstrate opposite patterns of brain activity during task performance.

It may also be the case that errors made by controls were related to processing deficits that had little to do with the inappropriate processing of distracters during the update cue, and were related to other factors that made them error prone (e.g., inattention at the probe). If this were the case the neural signature that would distinguish correct from incorrect trials may not have occurred in either prefrontal or striatal regions, and may have occurred at some other point during the trial than the update cue response period. Another possibility is that we simply lacked a sufficient number of error trials for controls to detect reliable differences of brain activity between correct and incorrect trials, given that patients' behavioral performance was significantly worse than controls for both Resist Distracter Lure and Update trial types. While it is difficult to make conclusive statements about the trial type accuracy results from our control sample, it was clear that patients demonstrated increased susceptibility to distracters, with a large effect size, and poorer updating behaviorally. Further, these performance deficits were associated with striatal and prefrontal activity during update cue presentation.

### Relationship between symptoms and brain activity

Our second goal was to identify a relationship between behavioral deficits of cognitive control, brain activity associated with these deficits, and symptom expression. Among patients, those individuals with higher striatal activity during incorrect trials had higher aberrant salience scores. Further, we found that within the same striatal region, the correlation between aberrant salience and incorrect trial activity was stronger than the correlation between aberrant salience and incorrect trial activity, suggesting some specificity, although the effect was only at trend level. When examining the relationship between striatal activity and other psychosis proneness scores we found that both correct and incorrect trial activity in the striatum similarly correlated with another psychosis proneness measure, but not with measures of anhedonia, providing some divergent validity for the construct of aberrant salience.

Results from the Update trial type analysis for patients revealed somewhat similar results, such that increases of globus pallidus activity during correct trial only were significantly correlated with aberrant salience and other measures of psychosis proneness and anhedonia, with left lateral globus pallidus selectively correlated with psychosis proneness and not anhedonia. For controls, we found negative correlations between correct and incorrect Update trial globus pallidus activity and aberrant salience, psychosis proneness, and anhedonia. Again, this was the opposite pattern that we observed for patients. If increased activity in the pallidum is associated filtering information into working memory storage (McNab and Klingberg, 2008) then the positive correlation between activity in the pallidum and symptom scores for patients makes sense as “loose” or increased filtering may contribute to symptomology the way aberrant salience does. However, it is not clear why pallidum activity would be correlated with measures of both psychosis proneness and anhedonia, nor is it clear why this pattern of correlations would differ for controls.

Our findings of a relationship between aberrant salience and dorsal striatal activity are somewhat distinct from previous studies. For example, Roiser et al. (2009) found that while some patients demonstrated adaptive motivational salience acquisition patients with greater delusional symptoms demonstrated aberrant salience acquisition. However, they found that aberrant salience was correlated only with negative symptoms. In another study examining aberrant salience of high risk for psychosis individuals, they found no group differences of aberrant reward prediction and no difference of dopamine synthesis capacity, but they did find aberrant salience acquisition differed behaviorally for the high-risk group and a positive correlation between ventral striatal responses and inappropriate salience assignment (Roiser et al., 2013).

As noted above, we found the opposite pattern of correlations between brain activity in and symptom scores for patients and controls. It is possible that this reflected reduced variance in symptom scores in controls vs. patients, as controls scored lower on the Chapman scales, with a similar trend for reduced scores on the ASI. However, in controls, aberrant salience was highly correlated with psychosis proneness but not anhedonia, providing evidence of discriminant validity within our nonclinical sample. Thus, while symptom expression for controls in this sample is lower than patients we have some evidence suggesting symptom expression within our control sample demonstrates some variability and behaves as expected. However, another potential interpretation of these findings is that the mechanisms that underlie psychosis proneness symptoms for non-psychotic individuals and psychotic individuals are different. Others work examining the relationship between brain activity and psychosis proneness of healthy individuals has found mixed results. For example, Ettinger et al. (2013) examined psychosis proneness of health participants and neural activation and found positive correlations between psychosis proneness and striatal and frontal regions, but previous work of theirs found negative relationships between brain activity in similar regions and psychosis proneness (from Ettinger et al., 2013; also see Corlett and Fletcher, 2012). Further work is needed to clarify some of the causal neural mechanisms of psychotic symptom expression within nonclinical samples and the degree to which similar relationships are found in clinical and non-clinical samples.

Taken together, these findings provide some evidence demonstrating that aberrant incentive salience is associated with psychosis and psychosis risk, and that as expected salience acquisition is associated with brain regions previously identified to be associated with this type of learning, including the ventral striatum. However, they acknowledge the conundrum that despite the relationship between the ventral striatum and motivational incentive salience acquisition, in schizophrenia the largest dopamine abnormality, thought to underlie deficits of salience acquisition, occurs in the dorsal rather than the ventral striatum (Howes et al., 2012). For patients the relationship between the dorsal striatum function, increased presynaptic dopamine storage and release with the dorsal striatum, aberrant salience and cognitive deficits associated with the disorder have been unclear. As such, the current study is the first to identify a relationship between dorsal striatal activity, cognitive control deficits associated with schizophrenia, and expression of aberrant salience symptoms.

### Limitations

Limitations of the study include the relatively modest sample size. It is possible that behavioral deficits we observed were the results of goal representation deficits associated with schizophrenia (reviewed in Barch and Ceaser, 2012). The impact of goal representation deficits, while not a focus of this current study, is something that warrants further exploration given that it may be a common mechanism of cognitive dysfunction for schizophrenia. Altered dopamine signaling was not measured in this study, and thus it is not clear to what extent our findings related to this dysfunction for patients with schizophrenia. Further work is needed to determine if, for example, aberrant salience symptoms correspond to changes of dopamine fluctuation or if it is associated with increased striatal activity for patients during distraction. This study also lacked a psychiatric control group. It may be the case that group differences we observed are also present when comparing individuals with schizoaffective, bi-polar disorder, or a mood disorder. Finally, it is possible that antipsychotic use by our patient participants may have influenced their results. Patient participants in this study were required to be stable on their medication for at least 2 weeks prior to study participation. We did not assess what medications patients were prescribed due to complexities with gathering this information that could result in, at best, inaccurate or, at worst, misleading information about medication use and its effect on our results. Antipsychotic medication use by our patient participants may have reduced aberrant salience symptoms and thus attenuated the relationship between symptom expression and brain activity we observed. However, without a direct measure of dopamine fluctuation it is difficult to say with, any certainty, what effect medication use by patients has had on our results.

## Conclusions

To our knowledge, this is the first study to find a relationship between dorsal striatal activity, cognitive control, and aberrant incentive salience, described by Kapur (2003), in patients with schizophrenia. We found evidence consistent with the hypothesis that the basal ganglia, particularly the associative striatum, may meaningfully contribute to the processing of cognitive control demands via information gating. Further, while we found evidence that both the striatum and DLPFC demonstrated altered activity during task demands, we found that for patients with schizophrenia striatal activity was selectively associated with the expression of aberrant salience symptoms, symptoms that are thought to result from dysregulated dopamine signaling. These findings provide potential treatment targets that could improve symptoms and functional outcome of patients with schizophrenia. For example, cognitive remediation that improves the regulation of information gating, a core component of executive control, an important predictor of functional outcome of severe mental illnesses (Martínez-Arán et al., 2002, 2007; Berk et al., 2013), should also impact aberrant salience symptom expression. Our future work will focus on further exploring the relationship between deficits of cognition associated with psychosis and brain functioning with the aim of developing more effective treatments for individuals with schizophrenia that will ultimately improve their quality of life.

### Conflict of interest statement

The authors declare that the research was conducted in the absence of any commercial or financial relationships that could be construed as a potential conflict of interest.
