# Supplementary material for: Striatal Activity is Associated with Deficits of Cognitive Control and Aberrant Salience for Patients with Schizophrenia
Source: Front Hum Neurosci. 2016 Feb 3;9:687. doi: 10.3389/fnhum.2015.00687 (PMC4738294; doi:10.3389/fnhum.2015.00687)
Supplement: Supplementary file 1 [file Table1.DOC]

# Supplementary Section A

A Breakdown of the Number of Trials for Each Task Condition and Trial Type

| **Upempty** | | **Upred** | | | **Upgreen** | | | |
| --- | --- | --- | --- | --- | --- | --- | --- | --- |
| 20 | | 48 | | | 52 | | | |
| **SMAIN** | **SMAINNP** | **RMAIN** | **RMAINUP** | **RMAINNP** | **UPDATE** | **UPDATEOP** | **UPDATENP** | **UPDATEOP2** |
| 14 | 6 | 20 | 20 | 8 | 20 | 20 | 8 | 4 |
