# Supplementary material for: Striatal Activity is Associated with Deficits of Cognitive Control and Aberrant Salience for Patients with Schizophrenia
Source: Front Hum Neurosci. 2016 Feb 3;9:687. doi: 10.3389/fnhum.2015.00687 (PMC4738294; doi:10.3389/fnhum.2015.00687)
Supplement: Supplementary file 2 [file Table2.DOC]

# Supplementary Section B

Regions Demonstrating an Effect of Diagnosis by Resist Distracter Lure Trial Type Accuracy within the Whole Brain

| **X** | **Y** | **Z** | **Size** | **Hemisphere** | **Region** | **BA** | **Effect at frames 8-12** | | | **Correct vs. Incorrect** | |
| --- | --- | --- | --- | --- | --- | --- | --- | --- | --- | --- | --- |
|  |  |  |  |  |  |  | *Analysis* | *F* | *p* | *Patients* | *Controls* |
| *Diagnosis* | |  |  |  |  |  |  |  |  |  |  |
| -43 | -6 | 34 | 105 | Left | Precentral Gyrus | 6 |  |  |  |  |  |
| -26 | -58 | 32 | 52 | Left | Angular Gyrus | 39 |  |  |  |  |  |
| -8 | 22 | 41 | 21 | Left | Cingulate Gyrus | 32 |  |  |  |  |  |
| 23 | -2 | 41 | 25 | Right | Middle Frontal Gyrus | 6 |  |  |  |  |  |
| -5 | 9 | 58 | 63 | Left | Superior Frontal Gyrus | 6 |  |  |  |  |  |
| 43 | -64 | -26 | 30 | Right Cerebellum | Tuber |  |  |  |  |  |  |
| *Accuracy* | |  |  |  |  |  |  |  |  |  |  |
| -30 | -59 | 39 | 29 | Left | Angular Gyrus | 39 |  |  |  |  |  |
| -34 | -26 | 54 | 59 | Left | Precentral Gyrus | 4 |  |  |  |  |  |
| *Diagnosis X Accuracy* | | |  |  |  |  |  |  |  |  |  |
| 34 | 14 | 38 | 23 | Right | Middle Frontal Gyrus | 8 | Dx X Acc | 21.56 | <0.001 | cor < incor | cor > incor |
| 47 | -10 | 45 | 25 | Right | Precentral Gyrus | 4 | Dx X Acc | 11.69 | 0.002 | cor < incor | cor > incor |
| *Accuracy X Time* | |  |  |  |  |  |  |  |  |  |  |
| 17 | -18 | 8 | 47 | Right | Thalamus |  |  |  |  |  |  |
| -11 | -21 | 12 | 54 | Left | Thalamus |  |  |  |  |  |  |
| 2 | 61 | 0 | 77 | Right | Middle Frontal Gyrus | 10 |  |  |  |  |  |
| -34 | 12 | 2 | 24 | Left | Insula | 13 |  |  |  |  |  |
| -50 | -16 | 14 | 129 | Left | Postcentral Gyrus | 43 |  |  |  |  |  |
| -33 | -14 | 11 | 22 | Left | Insula | 13 |  |  |  |  |  |
| -4 | -54 | 7 | 28 | Left | Posterior Cingulate | 30 |  |  |  |  |  |
| 10 | -68 | 28 | 59 | Right | Cuneus | 7 |  |  |  |  |  |
| -4 | -66 | 45 | 217 | Left | Precuneus | 7 |  |  |  |  |  |
| -3 | 26 | 34 | 21 | Left | Cingulate Gyrus | 32 |  |  |  |  |  |
| -39 | -26 | 55 | 58 | Left | Postcentral Gyrus | 3 |  |  |  |  |  |
| 5 | -51 | -1 | 57 | Right Cerebellum | Culmen |  |  |  |  |  |  |
| 17 | -65 | -16 | 56 | Right Cerebellum | Declive |  |  |  |  |  |  |
| *Diagnosis X Time* | |  |  |  |  |  |  |  |  |  |  |
| -22 | -7 | 28 | 21 | Left | Caudate |  |  |  |  |  |  |
| 0 | -33 | 9 | 36 | Right | Thalamus |  |  |  |  |  |  |
| 49 | -61 | -12 | 116 | Right | Fusiform Gyrus | 37 |  |  |  |  |  |
| 33 | -39 | -15 | 21 | Right | Fusiform Gyrus | 20 |  |  |  |  |  |
| -43 | -75 | -13 | 33 | Left | Fusiform Gyrus | 19 |  |  |  |  |  |
| -15 | 41 | -4 | 65 | Left | Anterior Cingulate | 32 |  |  |  |  |  |
| -41 | 44 | 11 | 26 | Left | Middle Frontal Gyrus | 10 |  |  |  |  |  |
| -37 | 32 | 18 | 25 | Left | Middle Frontal Gyrus | 46 |  |  |  |  |  |
| 46 | 19 | 24 | 33 | Right | Middle Frontal Gyrus | 46 |  |  |  |  |  |
| -11 | -22 | 70 | 88 | Left | Medial Frontal Gyrus | 6 |  |  |  |  |  |
| -35 | 42 | 30 | 57 | Left | Middle Frontal Gyrus | 9 |  |  |  |  |  |
| -25 | -64 | 31 | 37 | Left | Precuneus | 7 |  |  |  |  |  |
| 27 | -66 | 31 | 32 | Right | Precuneus | 7 |  |  |  |  |  |
| 39 | -42 | 50 | 196 | Right | Inferior Parietal Lobule | 40 |  |  |  |  |  |
| 27 | -15 | 63 | 115 | Right | Precentral Gyrus | 6 |  |  |  |  |  |
| -42 | 15 | 40 | 24 | Left | Precentral Gyrus | 9 |  |  |  |  |  |
| -36 | -45 | 47 | 43 | Left | Inferior Parietal Lobule | 40 |  |  |  |  |  |
| 0 | -72 | -13 | 23 | Left Cerebellum | Declive of Vermis |  |  |  |  |  |  |
| -24 | -80 | -25 | 35 | Left Cerebellum | Uvula |  |  |  |  |  |  |

Regions from the current data set that demonstrated diagnosis by Resist Update Lure accuracy. They are organized on the left side under headings like “Diagnosis” or “Accuracy” based on whether they demonstrated these effects when examining all 15 time frames of the trial. We only conducted follow up analyses for the update cue period on regions that demonstrated a significant interaction of accuracy and diagnosis. Statistics from the update cue response analysis can be found under the heading “Effect at frames 8-12”. “cor” = correct trials and “incor” = incorrect trials. **p*<0.05 and ***p*<0.01, uncorrected. “no diff” signifies no statistically significant difference.
