# Supplementary material for: Striatal Activity is Associated with Deficits of Cognitive Control and Aberrant Salience for Patients with Schizophrenia
Source: Front Hum Neurosci. 2016 Feb 3;9:687. doi: 10.3389/fnhum.2015.00687 (PMC4738294; doi:10.3389/fnhum.2015.00687)
Supplement: Supplementary file 3 [file Table3.DOC]

# Supplementary Section C

**Regions Demonstrating an Effect of Diagnosis by Update Trial Type Accuracy with the Whole Brain**

| **X** | **Y** | **Z** | **Size** | **Hemisphere** | **Region** | **BA** | **Effect at frames 8-12** | | | **Correct vs. Incorrect** | |
| --- | --- | --- | --- | --- | --- | --- | --- | --- | --- | --- | --- |
|  |  |  |  |  |  |  | *Analysis* | *F* | *p* | *Patients* | *Controls* |
| *Diagnosis* |  |  |  |  |  |  |  |  |  |  |  |
| 1 | -16 | 6 | 912 | Right | Thalamus |  |  |  |  |  |  |
| -45 | 23 | 6 | 239 | Left | Inferior Frontal Gyrus | 45 |  |  |  |  |  |
| 25 | 28 | -2 | 25 | Right | Inferior Frontal Gyrus | 47 |  |  |  |  |  |
| -30 | 20 | 3 | 50 | Left | Insula | 13 |  |  |  |  |  |
| 32 | 15 | 4 | 46 | Right | Insula | 13 |  |  |  |  |  |
| -46 | -4 | 33 | 204 | Left | Precentral Gyrus | 6 |  |  |  |  |  |
| 49 | -11 | 23 | 28 | Right | Precentral Gyrus | 6 |  |  |  |  |  |
| 0 | -29 | 29 | 33 | Left | Cingulate Gyrus | 23 |  |  |  |  |  |
| -27 | -63 | 29 | 30 | Left | Precuneus | 7 |  |  |  |  |  |
| -39 | -46 | 41 | 81 | Left | Inferior Parietal Lobule | 40 |  |  |  |  |  |
| -4 | 12 | 56 | 283 | Left | Superior Frontal Gyrus | 6 |  |  |  |  |  |
| -30 | -1 | 52 | 33 | Left | Middle Frontal Gyrus | 6 |  |  |  |  |  |
| 1 | -29 | 67 | 66 | Right | Paracentral Lobule | 6 |  |  |  |  |  |
| -6 | -21 | -13 | 54 | Left Brainstem | Red Nucleus |  |  |  |  |  |  |
| -28 | -40 | -43 | 32 | Left Cerebellum | Cerebellar Tonsil |  |  |  |  |  |  |
| 33 | -69 | -45 | 38 | Right Cerebellum | Inferior Semi-Lunar Lobule |  |  |  |  |  |  |
| -29 | -70 | -46 | 52 | Left Cerebellum | Inferior Semi-Lunar Lobule |  |  |  |  |  |  |
| *Diagnosis X Accuracy* | | |  |  |  |  |  |  |  |  |  |
| -20 | -21 | 2 | 39 | Left | Thalamus |  | Dx X Acc | 10.98 | 0.002 | no diff | cor < incor** |
| 13 | -25 | 2 | 25 | Right | Thalamus |  | Dx X Acc | 8.63 | 0.006 | no diff | cor < incor* |
| -5 | -27 | -11 | 27 | Left Brainstem | Red Nucleus |  | Dx X Acc | 5.43 | 0.03 | cor > incor* | cor < incor* |
| *Accuracy X Time* | |  |  |  |  |  |  |  |  |  |  |
| 0 | 42 | -5 | 36 | Left | Anterior Cingulate | 32 |  |  |  |  |  |
| -44 | 10 | 30 | 21 | Left | Inferior Frontal Gyrus | 9 |  |  |  |  |  |
| -36 | -31 | 43 | 57 | Left | Inferior Parietal Lobule | 40 |  |  |  |  |  |
| 23 | 24 | 40 | 59 | Right | Middle Frontal Gyrus | 8 |  |  |  |  |  |
| 25 | -4 | 44 | 56 | Right | Middle Frontal Gyrus | 6 |  |  |  |  |  |
| -27 | 1 | 50 | 124 | Left | Middle Frontal Gyrus | 6 |  |  |  |  |  |
| -24 | -43 | -9 | 33 | Left | Parahippocampal Gyrus | 36 |  |  |  |  |  |
| -20 | -70 | 42 | 59 | Left | Precuneus | 7 |  |  |  |  |  |
| -3 | 15 | 55 | 203 | Left | Superior Frontal Gyrus | 6 |  |  |  |  |  |
| 36 | -41 | 9 | 30 | Right | Superior Temporal Gyrus | 41 |  |  |  |  |  |
| *Diagnosis X Time* | |  |  |  |  |  |  |  |  |  |  |
| -8 | -70 | 32 | 33 | Left | Cuneus | 7 |  |  |  |  |  |
| 52 | -59 | -18 | 27 | Right | Fusiform Gyrus | 37 |  |  |  |  |  |
| -40 | 44 | 3 | 124 | Left | Inferior Frontal Gyrus | 10 |  |  |  |  |  |
| -35 | -45 | 45 | 21 | Left | Inferior Parietal Lobule | 40 |  |  |  |  |  |
| 1 | -92 | -14 | 55 | Right | Lingual Gyrus | 18 |  |  |  |  |  |
| 0 | 38 | 40 | 31 | Left | Medial Frontal Gyrus | 8 |  |  |  |  |  |
| -3 | -26 | 65 | 26 | Left | Medial Frontal Gyrus | 6 |  |  |  |  |  |
| -43 | 8 | 47 | 48 | Left | Middle Frontal Gyrus | 6 |  |  |  |  |  |
| -55 | 11 | 9 | 23 | Left | Precentral Gyrus | 44 |  |  |  |  |  |
| -54 | -3 | 20 | 23 | Left | Precentral Gyrus | 6 |  |  |  |  |  |
| 42 | 17 | 35 | 22 | Right | Precentral Gyrus | 9 |  |  |  |  |  |
| 29 | -67 | 29 | 39 | Right | Precuneus | 19 |  |  |  |  |  |
| 36 | -42 | 37 | 23 | Right | Sub-Gyral | 40 |  |  |  |  |  |
| -6 | 12 | 60 | 120 | Left | Superior Frontal Gyrus | 6 |  |  |  |  |  |
| -48 | -66 | -16 | 30 | Left Cerebellum | Declive |  |  |  |  |  |  |
| *Diagnosis X Accuracy X Time* | | |  |  |  |  |  |  |  |  |  |
| 25 | 42 | 5 | 23 | Right | Medial Frontal Gyrus | 10 | Dx X Acc | 2.33 | 0.14 |  |  |

Regions within our anatomical masks from the current data set that demonstrated diagnosis by Update accuracy. They are organized on the left side under headings like “Diagnosis” or “Accuracy” based on whether they demonstrated these effects when examining all 15 time frames of the trial. We only conducted follow up analyses for the update cue period on regions that demonstrated a significant interaction of accuracy and diagnosis. Statistics from the update cue response analysis can be found under the heading “Effect at frames 8-12”. “cor” = correct trials and “incor” = incorrect trials. **p*<0.05 and ***p*<0.01, uncorrected. “no diff” signifies no statistically significant difference.
