# Supplementary material for: Striatal Activity is Associated with Deficits of Cognitive Control and Aberrant Salience for Patients with Schizophrenia
Source: Front Hum Neurosci. 2016 Feb 3;9:687. doi: 10.3389/fnhum.2015.00687 (PMC4738294; doi:10.3389/fnhum.2015.00687)
Supplement: Supplementary file 4 [file DataSheet1.DOC]

# Supplementary Section D

**Replication fMRI Data Analysis**

*Types of GLMS*

The GLMs used for our replication analyses focused on the 3 events that occurred during the update cue of the trial. We refer to this as the “*Condition*” analysis. For the Condition analysis all trial types were collapsed into three events, ignoring the different probe types that could occur within a condition: 1) the update condition (Upgreen) in which participants were required to make a update of information, 2) the interference control condition (Upred) in which participants were required to ignore distracters, and 3) the maintenance condition (Upempty) in which participants were not presented any shape stimuli, and were simply required to maintain the items from the memory set. Analyses at the level of Condition could reveal differences between groups when tasked with making an update, ignoring distracters, or simply maintain information.

*Analysis Approach: Independently Defined ROIs versus Anatomical Mask*

One approach we used to examine the effects of task Condition was to use ROIs identified in a previous study of healthy controls using the same task paradigm to assess the brain activity as a function of task condition (Ceaser et al., under review). The results of this study revealed patterns of brain activity in response to task conditions that differed between cortical and subcortical brain regions. Using these independently defined ROIs allows us to enhance statistical power to detect effects by restricting our analysis to only voxels within the previously defined regions, but also to attempt to replicate our previous findings and to determine whether activity in these regions went on to interact with diagnosis. These regions will be referred to as the *independently defined ROIs* in the results section. We considered a region to be significant for this analyses if *p*<.05.

We were also specifically interested in whether regions in the prefrontal cortex and basal ganglia, specifically the dorsal striatum, demonstrated condition effects. Thus, as a second approach we used anatomical masks of the basal ganglia (Wang et al., 2008) and the prefrontal cortex (Rajkowska & Goldman-Rakic, 1995), and examined voxel-wise analyses of brain activity within these masks. These ROIs were combined into a single mask and we used a small volume type I error correction, implemented via the Analysis of Functional Neuroimages AlphaSim, of *Z >* 2.32, *k* = 20 voxels for this combined ROI mask. This analysis could produce regions that show main effects of condition and time, and a 2-way interaction of condition by time.

*Replication of Prior Results in Healthy Individuals*

To determine whether we could replicate findings from our previous study in the Independently defined ROIs, we examined only the healthy participants in the current study and conducted a repeated measures ANOVA at the region level with condition (3 levels; Upgreen, Upred, and Upempty) and time (5 levels) as factors. The 5 TRs we used for this analysis corresponded to the 5 TR following the presentation of the update cue (frames 8-12, accounting for hemodynamic lag). Only correct trials were examined in this analysis. If an ROI demonstrated either a significant effect of condition or an interaction of condition and time, we then conducted follow-up analyses to determine the source of theses effect, with each analyses comparing one condition to another. Separate ANOVAs were done for conditions Upgreen versus Upempty and Upred versus Upempty.

Following the ROI analysis using previously defined regions, next conducted voxel-wise analyses within our anatomical masks using only the controls to see if our previous results would replicate. Specifically, we conducted voxel-wise repeated measures ANOVA with condition (3 levels; Upgreen, Upred, and Upempty) and time (15 levels) as factors. We included all 15 frames of the trial in the analysis to capture regions that show effects of interest, but at *any time* during the course of the trial. We then tested whether or not condition effects occurred when we would expect them to during the update component of the trials. For any region demonstrating an effect of condition or an interaction of condition and time, we followed up with an analysis that focused on 5 frames that occurred after the presentation of the update cues and prior to the response of the probe (frames 8-12, accounting for hemodynamic lag). Specifically, we conduced a second repeated measures ANOVA at the region level with condition (3 levels) and time (5 levels) as factors. We examined the source of any such effect in the manner described above, by comparing Upgreen versus Upempty and Upred versus Upempty separately.

*Condition Analysis of Controls and Patients*

We first examined whether the striatal activity of patients displayed different responses to task Condition, updating (Upgreen), interference control (Upred) and maintenance (Upempty) demands, when compared with controls. For the independently defined ROIs, we conducted a repeated measures ANOVA at the region level with condition (3 levels; Upgreen, Upred, and Upempty), diagnosis (2 levels; patients and controls), and time (5 levels; frames 8-12) as factors, using only the data from correct trials. Because we were only interested in regions that interacted with at least condition and diagnosis, we only explored the effects of regions that showed a significant 2-way interaction of condition by diagnosis, or a significant 3-way interaction of condition by time by diagnosis.

We then conducted voxel-wise analyses within our *a priori* anatomical masks. This analysis involved a repeated measures ANOVA with diagnosis (2 levels), update cue (3 levels), and time (15 frames) as factors, treating subjects as a random factor. Given that we were specifically interested in regions that interacted with both diagnosis and condition, regions that demonstrated either a significant 2-way interaction of diagnosis by condition or a 3-way interaction of diagnosis by time by condition were used for further analyses. Of note, we recognize that should we find results in regions in our a priori mask and not regions outside the mask, we cannot claim specificity to regions inside the mask given the differential levels of significance required. However, we felt that this was the best balance between providing sufficient power to test a priori hypotheses and being open to unpredicted effects.

We included all 15 frames in the analyses in the previous step to capture regions that show effects of interest, but at *any time* during the course of the trial. Once these regions were identified, we then tested whether condition effects in these regions occurred in response to the update events, using a repeated measures ANOVA with diagnosis (2 levels), update cue (3 levels), and time (5 levels; frames 8-12) as factors, treating subjects as a random factor. Only correct trials were used for this analysis. For regions demonstrating significant interactions of interest, we explored the interaction in the manner described above examining Upgreen versus Upempty and Upred versus Upempty for both diagnostic groups separately.

**Replication fMRI Data Results**

*Independently Defined ROI Results*

Examination of brain activity in healthy controls within the independently defined ROIs demonstrated either an effect of condition or an interaction of condition by time (Replication Table 1) in all seven regions. This included bilateral middle frontal gyrus (MFG), left lateral inferior frontal gyrus (IFG), right lateral precentral gyrus, left lateral putamen, and right lateral caudate body (Replication Table 1). Previously we found that these regions demonstrated either effects of condition or interactions of condition and time, suggesting these regions were sensitive to task condition. In the prior study when examining the pattern of activity in these regions in response to the presentation of the update cue, we found that all regions demonstrated significant differences between Upgreen and Upempty conditions, suggesting that activity in both cortical and subcortical regions demonstrated robust activity to updating demands when compared with activity during simple maintenance. Only cortical regions, however, demonstrated either significant or trend level differences between Upred and Upempty, suggesting striatal activity selectively activated to updating demands when compared with distracter presentation and simple maintenance. In the current study, as predicted, Upgreen activity for all cortical regions was significantly greater than Upempty activity (with the exception of one region in the precentral gyrus, although it trended towards significance). Only one cortical region demonstrated a predicted significant difference between Upred and Upempty (IFG, -39, 4, 30; Replication Table 1 and Replication Figure 1A), such that Upred activity was greater than Upempty activity. This finding is a replication of our previous study, suggesting that activity in the IFG is sensitive to both updating and distracter presentation task demands. Other regions, previously found to sensitive to both task demands (i.e. right lateral precentral gyrus and left lateral MFG) did not demonstrate the same condition sensitivity in the current sample, suggesting that condition sensitivity may be localized to the IFG. Of the striatal regions, the region in the left putamen demonstrated significantly greater Upgreen versus Upempty activity, but there was no difference between Upred and Upempty (-18, -3, 13; Replication Table 1 and Replication Figure 1C). Thus, we again found that regions within the caudate and putamen demonstrated condition sensitivity to Upgreen relative to Upempty and not Upred relative to Upempty. This is consistent with theproposed role of the striatum as an information gate, striatal activity activates when the gate is open but not when distracters a presented. Further, while a region in the right lateral caudate demonstrated a significant interaction of condition and time, neither Upgreen nor Upred activity significantly differed from Upempty activity. When examining the time course of this region Upempty activity was, unexpectedly, intermediate to that of Upgreen and Upred, which may explain why neither condition differed from Upempty. When we examined whether Upred and Upgreen significantly differed within this caudate region we found that they did such that Upgreen was significantly greater than Upred (*F*(1,19) = 4.85, *p* = 0.02).

*Anatomical Mask of Basal Ganglia and Prefrontal Cortex*

We next conducted voxel-wise analyses in our anatomical *a priori* regions of interest using only the data from the healthy controls. Regions demonstrating an effect of condition or an interaction of condition by time from healthy control subjects can be found in Replication Table 2. One region demonstrated a main effect of condition (left MFG, -43, 29, 27), with follow-up analysis indicating a highly significant effect of condition during frames 8-12 (*F*(2,19) = 18.92, *p* < 0.001; Replication Figure 2A), such that Upgreen was significantly greater than Upempty but there was no difference between Upred and Upempty. There were 5 regions that demonstrated

Replication Table 1: Regions from the Previous Data Set and Their Condition Effect in Healthy Controls from the Current Data Set

| **X** | **Y** | **Z** | **Size** | **Hemisphere** | **Region** | **BA** | **Effect at frames 8-12** | | | **Direction** | |
| --- | --- | --- | --- | --- | --- | --- | --- | --- | --- | --- | --- |
|  |  |  |  |  |  |  | *Analysis of Current Study* | *F* | *p* | *Upgreen vs. Upempty* | *Upred vs. Upempty* |
| **Controls, Independently Defined Regions** | | | | |  |  |  |  |  |  |  |
| *Condition Effect in Previous Study* | | | | |  |  |  |  |  |  |  |
| -43 | 22 | 30 | 27 | Left | MFG | 9 | Cond | 6.83 | 0.003 | G > E** | no diff |
| -39 | 4 | 30 | 25 | Left | IFG | 9 | Cond | 12.89 | <0.0001 | G > E** | R > E* |
| 41 | 5 | 33 | 20 | Right | Precentral Gyrus | 9 | Cond X Time | 3.42 | 0.03 | no diff | no diff |
| *Condition X Time in Previous Study* | | | | |  |  |  |  |  |  |  |
| -18 | -3 | 13 | 155 | Left | Putamen |  | Cond | 4.72 | 0.02 | G > E* | no diff |
| 13 | -10 | 19 | 46 | Right | Caudate Body |  | Cond X Time | 4.37 | 0.01 | no diff | no diff |
| -42 | 17 | 29 | 211 | Left | MFG | 9 | Cond | 8.84 | 0.001 | G > E** | no diff |
| 42 | 13 | 32 | 79 | Right | MFG | 9 | Cond X Time | 3.54 | 0.02 | G > E** | no diff |

Independently defined ROIs are listed in the table under the heading “Controls, Independently Defined Regions” and are organized based on whether they demonstrated an effect of condition or interaction of condition and time in the previous study. Statistics from the update cue response analysis can be found under the heading “Effect at frames 8-12”. Listed under this heading is what analysis the independently defined ROIs demonstrated the effect as well as the corresponding F and p values of that effect. In the table, under the heading Direction, the pattern and significance of that effect is listed. MFG = Middle Frontal Gyrus and IFG=Inferior Frontal Gyrus. G = Upgreen trials, E = Upempty trials, and R = Upred trials. **p*<0.05 and ***p*<0.01. “no diff” signifies no statistically significant difference.

significant interactions of condition and time when examining all 15 timepoints, including 3 regions in the MFG, bilaterally. Only 2 of them (-42, 24, 23 and 42, 21, 29) demonstrated significant effects of condition or condition by time in response to the update cue (Replication Table 2). For both of these regions, Upgreen activity was significantly greater than Upempty, but neither showed differences between Upred and Upempty activity. While there were two regions in the dorsal striatum that demonstrated interactions of condition and time when examining all 15 frames of the trial, only a region in the caudate (-12, 7, 9) continued to demonstrated a significant interaction of condition and time in analyses restricted to the time periods associated with the update cue (*F*(2,19) = 2.99, *p* = 0.004; Replication Table 2). For this region Upgreen activity was significantly greater than Upempty activity, and there was a significant difference between Upred and Upempty, such that, unexpectedly, Upempty activity was significantly greater than Upred activity (Replication Figure 2B). Thus, a number of the regions identified in a previous study demonstrated predicted differences between Upgreen and the comparison condition, Upempty, such that Upgreen activity was greater than Upempty. Only one region in the IFG demonstrated a predicted difference between Upred and Upempty.

*Condition Results for Both Patients and Controls*

*Independently Defined ROIs*

We started by examining whether activity in the independently defined ROIs differed as a function of diagnostic group. Results from this analysis can be found in Replication Table 3. We found that activity within 2 regions demonstrated a significant interaction of diagnosis and condition, including a region in the left IFG (-39, 4, 30) and a region in the left MFG (-42, 17, 29). For the IFG, activity during the Upgreen condition was significantly greater than activity during Upempty for both patients and controls (Replication Figure 1A and 4.2B). Controls demonstrated significantly greater Upred than Upempty activity (there was a trend towards greater Upred versus Upempty for patients, *p* = 0.095). When we compared Upgreen activity for the IFG (-39, 4, 30) between groups we found a trend towards significantly greater for controls

Replication Figure 1: Brain Activity of Healthy Controls and Patients Within Regions Defined in a Previous Data Set


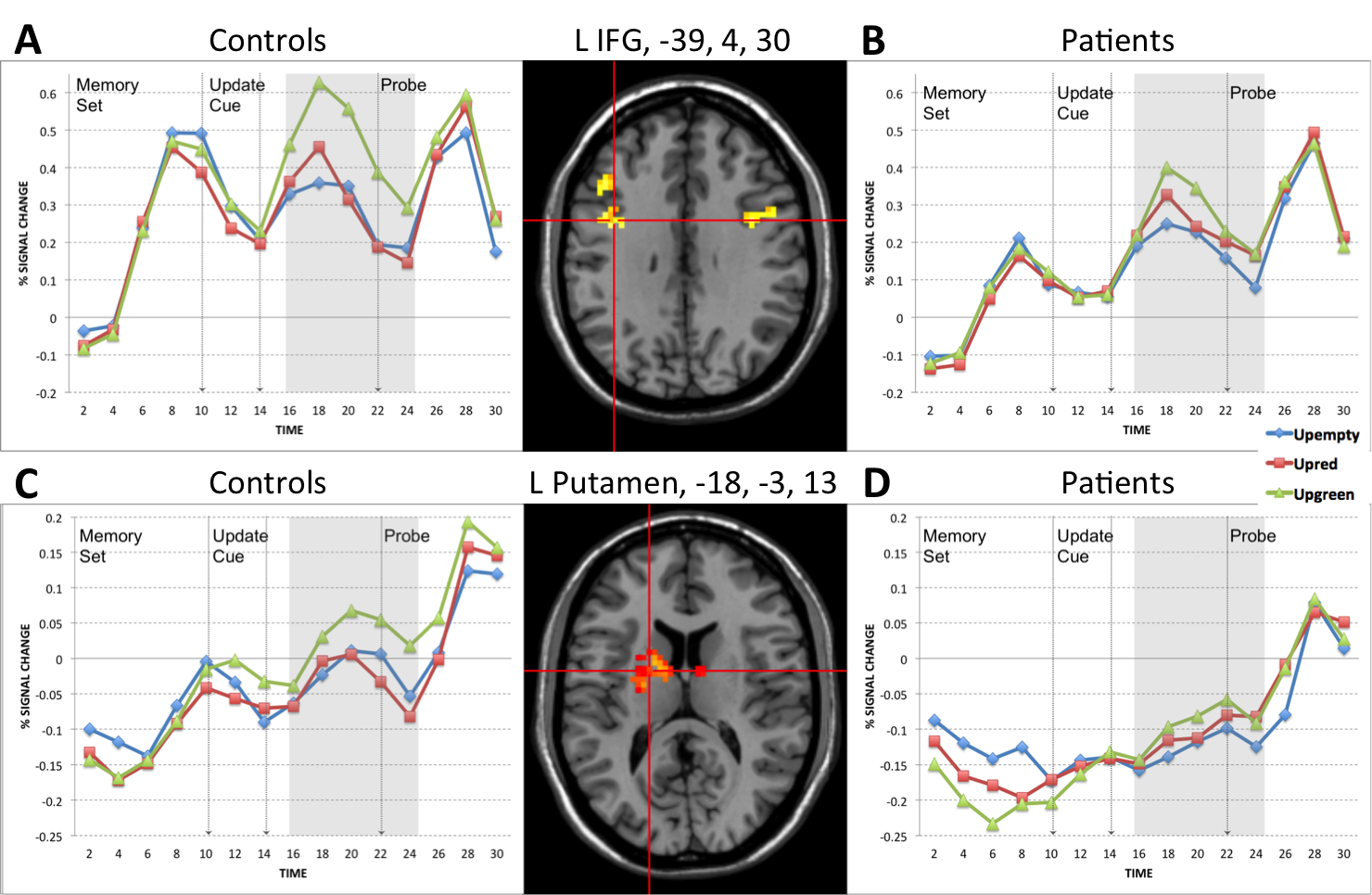


Replication Table 2: Regions from the Current Data Set of Healthy Controls that Demonstrated Effects of Condition

| **X** | **Y** | **Z** | **Size** | **Hemisphere** | **Region** | **BA** | **Effect at frames 8-12** | | | **Direction** | |
| --- | --- | --- | --- | --- | --- | --- | --- | --- | --- | --- | --- |
|  |  |  |  |  |  |  | *Analysis* | *F* | *p* | *Upgreen vs. Upempty* | *Upred vs. Upempty* |
| **Regions Identified in Current Data Set in Healthy Controls** | | | | | |  |  |  |  |  |  |
| *Condition* | |  |  |  |  |  |  |  |  |  |  |
| -43 | 29 | 27 | 32 | Left | MFG | 9 | Cond | 18.92 | <0.0001 | G > E** | no diff |
| *Condition X Time* | | |  |  |  |  |  |  |  |  |  |
| -12 | 7 | 9 | 36 | Left | Caudate Body |  | Cond X Time | 2.99 | 0.004 | G > E** | E > R* |
| 20 | -1 | 9 | 21 | Right | Putamen |  | Cond X Time | 0.56 | 0.81 |  |  |
| 37 | 51 | 3 | 47 | Right | MFG | 10 | Cond X Time | 1.11 | 0.41 |  |  |
| -42 | 24 | 24 | 285 | Left | MFG | 46 | Cond X Time | 3.02 | 0.04 | G > E* | no diff |
| 42 | 21 | 29 | 262 | Right | MFG | 9 | Cond | 8.4 | 0.001 | G > E** | no diff |

Regions within our anatomical masks from healthy control in the current data set that demonstrated Condition effects are listed in the table under the heading “Regions Identified in the Current Data Set in Healthy Controls”, and are organized on the left side under headings like “Diagnosis” or “Condition X Time” based on whether they demonstrated these effects when examining all 15 time frames of the trial. Listed under the heading “Effect at frames 8-12” is in what analysis the independently defined ROIs demonstrated an effect as well as the corresponding F and p values of that effect. In the table, under the heading Direction, the pattern and significance of that effect is listed. MFG = Middle Frontal Gyrus. G = Upgreen trials, E = Upempty trials, and R = Upred trials. **p*<0.05 and ***p*<0.01. “no diff” signifies no statistically significant difference.

Replication Figure 2: Regions of Healthy Controls from the Current Data Set that Demonstrated Effects of Condition


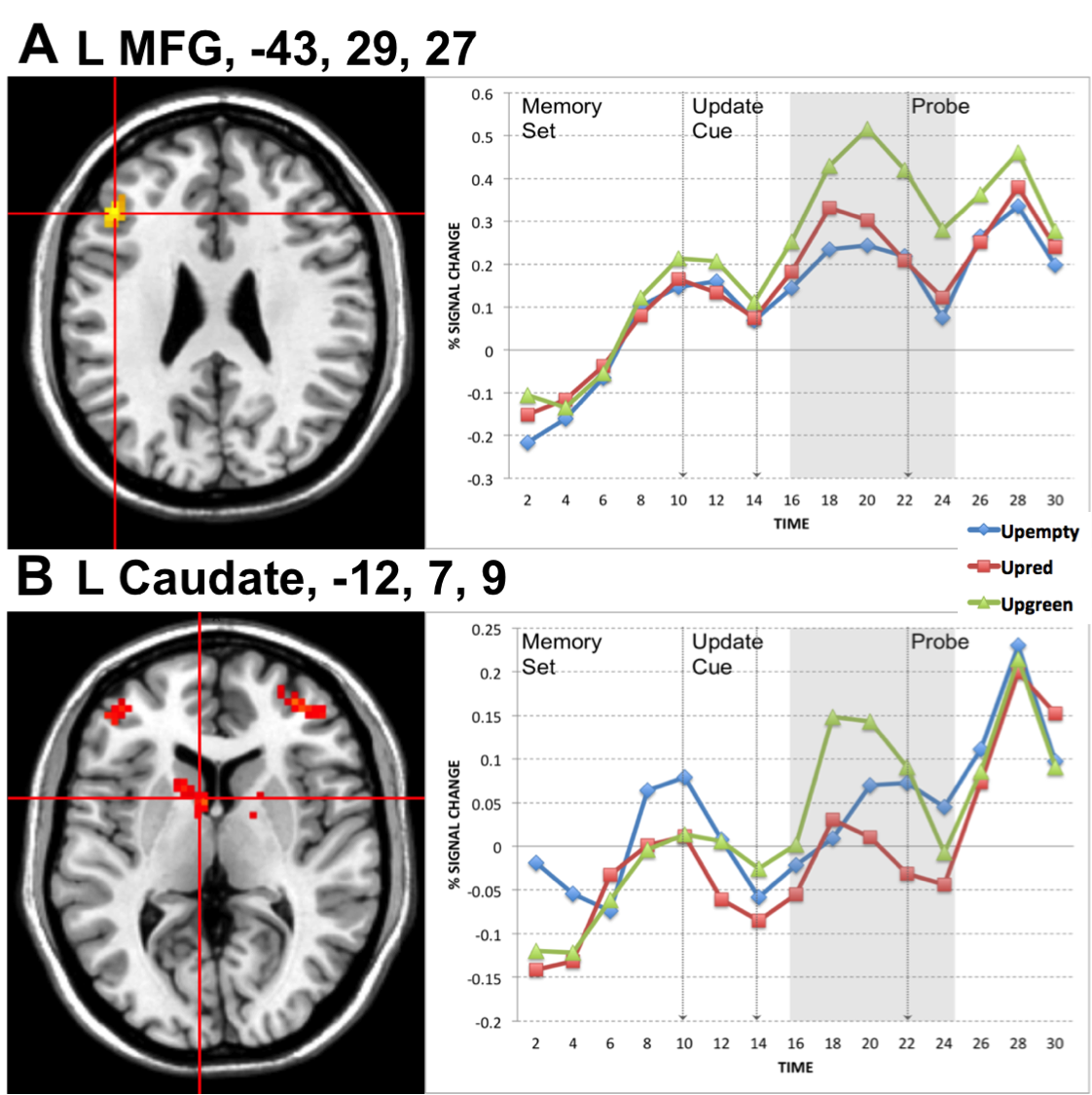


than it was for patients (*F*(1,41) = 3.13, *p* = 0.09), but no difference between groups for Upred activity (*F*(1,41) = 0.18, *p* = 0.67).The other region that demonstrated a diagnosis by condition interaction was a region in the left MFG. In this region controls demonstrated significantly greater Upgreen than Upempty activity and this difference trended towards significance for patients (*p* = 0.07). Interestingly, within this region, patients, but not controls showed greater Upred than Upempty activity. When we compared Upgreen activity for the MFG (-42, 17, 29) between groups we found that activity was significantly greater for controls than it was for patients (*F*(1,41) = 4.52, *p* = 0.04), but no difference between groups for Upred activity (*F*(1,41) = 0.52, *p* = 0.47).

A region in the right caudate body (13, -10, 19) demonstrated a 3-way interaction of diagnosis by time by condition (Replication Table 3). However, when comparing Upgreen versus Upempty and Upred versus Upempty during the frames following the update cue, neither diagnostic group demonstrated a significant difference between conditions. As mentioned above, examining the time course for this region revealed an unexpected increase of Upempty activity, such that it activity was intermediate to Upgreen and Upred for controls. This was not also the case for patients. Generally, putamen activity for patients was numerically lower than controls and didnot appear to respond to trial events (i.e. the memory set and update cue) that way that control putamen activity did. The elevated Upempty activity and Upgreen activity for controls may explain why we observed a significant interaction of diagnosis by condition for this region whilst failing to observe differences between the comparison condition and Upgreen/Upred. However, when we compared caudate activity between diagnostic groups during Upgreen and Upred conditions we found that while controls had numerically higher Upgreen and Upred activity, these differences were not significant (*F*(1,41) = 2.19, *p* = 0.15 and *F*(1,41) = 1.26, *p* = 0.27, respectively).

*Anatomical Mask of Basal Ganglia and Prefrontal Cortex*

Replication Table 3: Regions from a Previous Data Set and Their Diagnosis by Condition Effects for Patients and Controls from the Current Data Set

| **X** | **Y** | **Z** | **Size** | **Hemi** | **Region** | **BA** | **Diagnosis Interaction** | | | **Direction (Patients)** | | **Direction (Controls)** | |
| --- | --- | --- | --- | --- | --- | --- | --- | --- | --- | --- | --- | --- | --- |
|  |  |  |  |  |  |  | *Analysis* | *F* | *p* | *Upgreen vs. Upempty* | *Upred vs. Upempty* | *Upgreen vs. Upempty* | *Upred vs. Upempty* |
| *Condition* | |  |  |  |  |  |  |  |  |  |  |  |  |
| -43 | 22 | 30 | 27 | Left | MFG | 9 | Dx X Cond | 2.55 | 0.09 |  |  |  |  |
| -39 | 4 | 30 | 25 | Left | IFG | 9 | Dx X Cond | 3.76 | 0.03 | G > E** | no diff | G > E** | R > E* |
| 41 | 5 | 33 | 20 | Right | Precentral Gyrus | 9 | Dx X Cond | 0.56 | 0.57 |  |  |  |  |
| *Condition X Time* | | |  |  |  |  |  |  |  |  |  |  |  |
| -18 | -3 | 13 | 155 | Left | Putamen |  | Dx X Cond | 1.51 | 0.28 |  |  |  |  |
| 13 | -10 | 19 | 46 | Right | Caudate Body |  | 3-way | 2.08 | 0.03 | no diff | no diff | no diff | no diff |
| -42 | 17 | 29 | 211 | Left | MFG | 9 | Dx X Cond | 3.54 | 0.02 | no diff | R > E* | G > E** | no diff |
| 42 | 13 | 32 | 79 | Right | MFG | 9 | Dx X Cond | 2.54 | 0.09 |  |  |  |  |

Statistics for independently defined regions that demonstrated diagnosis by Condition effects. We only conducted follow up analyses for the update cue period on regions that demonstrated a significant effect of condition or a significant interaction of condition and time. Statistics from the update cue response analysis can be found under the heading “Effect at frames 8-12”. The direction of Upgreen and Upred versus Upempty effects for controls found in the previous analysis (Replication Table 1) are listed to the right of the direction of effects for patients to ease comparison between the two groups within this table. G = Upgreen trials, E = Upempty trials, and R = Upred trials. **p*<0.05 and ***p*<0.01. “no diff” signifies no statistically significant difference.

Regions from our *a priori* anatomical mask analysis that demonstrated either a main effects of task condition, time, and diagnosis, or interactions are listed in Replication Table 4. We focused our analysis on regions that demonstrated either an interaction of diagnosis by condition or Diagnosis by time by condition. Only one region demonstrated a condition X diagnosis interaction: left lateral MFG (-41, 30, 26; not in Replication Table 4), an interaction that held when examining only the 5 frames following the update cue (*F*(2,41) = 6.4, *p* = 0.004). The pattern of activity within this region was that only controls demonstrated significant greater activity during Upgreen versus Upempty (Replication Table 4, Replication Figure 3), with no significant difference between Upred and Upempty. When we compared differences of Upgreen and Upred activity within this region between groups we found that controls has significantly greater activity during Upgreen relative to patients (*F*(1,41) = 4.53, *p* = 0.04) but there was no difference between Upred activity (*F*(1,41) = 0.7, *p* = 0.41).

**Replication Discussion**

## 5.1. Replication of Prior fMRI Results in Healthy Individuals:

### 5.1.1. Independently Defined ROI

Previously we found that cortical activity increased during both information updating events and distracter presentation compared to a basic maintenance condition, but that activity within the dorsal striatum selectively activated to information updating and not interference control (Ceaser et al., in prep). Further, we found that during the presentation of the update cue, only brain activity within an anatomical mask of the basal ganglia, and not the prefrontal or parietal cortices, could significantly predicted whether an individual made a correct or incorrect response at the probe. These results provided some support for the idea that the basal ganglia may function as a mechanism of information gating during cognitive control, and were consistent with previous studies examining subcortical contributions to cognitive control. For

Replication Table 4: Regions from the Current Data Set that Demonstrated Diagnosis by Condition Effects Within our Anatomical Masks

| **X** | **Y** | **Z** | **Size** | **Hemisphere** | **Region** | **BA** |
| --- | --- | --- | --- | --- | --- | --- |
| *Diagnosis* | |  |  |  |  |  |
| -23 | -10 | 4 | 149 | Left | Globus Pallidus |  |
| 24 | -11 | 5 | 126 | Right | Globus Pallidus |  |
| 24 | -11 | 5 | 126 | Right | Globus Pallidus |  |
| 16 | -1 | 19 | 34 | Right | Caudate Body |  |
| 16 | -1 | 19 | 34 | Right | Caudate Body |  |
| -40 | 38 | 7 | 282 | Left | IFG | 46 |
| 29 | 53 | 2 | 57 | Right | MFG | 10 |
| -36 | 20 | 23 | 33 | Left | MFG | 9 |
| 40 | 9 | 30 | 65 | Right | IFG | 9 |
| 30 | 30 | 28 | 30 | Right | MFG | 9 |
| -40 | 8 | 33 | 31 | Left | Precentral Gyrus | 9 |
| *Condition* | |  |  |  |  |  |
| -42 | 4 | 31 | 22 | Left | IFG | 9 |
| *Condition X Time* | | |  |  |  |  |
| -19 | 0 | 8 | 243 | Left | Putamen |  |
| 17 | 3 | 10 | 152 | Right | Putamen |  |
| 36 | 52 | 5 | 40 | Right | MFG | 10 |
| -41 | 23 | 25 | 390 | Left | MFG | 46 |
| 42 | 20 | 29 | 265 | Right | MFG | 9 |
| -28 | 34 | 33 | 21 | Left | SFG | 9 |
| *Diagnosis X Time* | | |  |  |  |  |
| -24 | -4 | 2 | 53 | Left | Putamen |  |
| -16 | -5 | 20 | 24 | Left | Caudate Body |  |
| 15 | -6 | 21 | 35 | Right | Caudate Body |  |
| -39 | 5 | 31 | 39 | Left | IFG | 9 |
| 28 | 31 | -5 | 37 | Right | IFG | 47 |
| -39 | 43 | 6 | 190 | Left | MFG | 46 |
| -39 | 34 | 25 | 44 | Left | MFG | 46 |
| 46 | 22 | 26 | 63 | Right | MFG | 46 |
| 35 | 6 | 32 | 22 | Right | Precentral Gyrus | 9 |

Regions within our anatomical masks from the current data set that demonstrated diagnosis by Condition effects within our anatomical masks. They are organized on the left side under headings like “Diagnosis” or “Condition X Time” based on whether they demonstrated these effects when examining all 15 time frames of the trial. We only conducted follow up analyses for the update cue period on regions that demonstrated a significant interaction of condition and diagnosis. Statistics from the update cue response analysis can be found under the heading “Effect at frames 8-12”. G = Upgreen trials, E = Upempty trials, and R = Upred trials. **p*<0.05 and ***p*<0.01. “no diff” signifies no statistically significant difference.

Replication Figure 3: Frontal Region from the Current Data Set that Demonstrated a Diagnosis by Condition Interaction


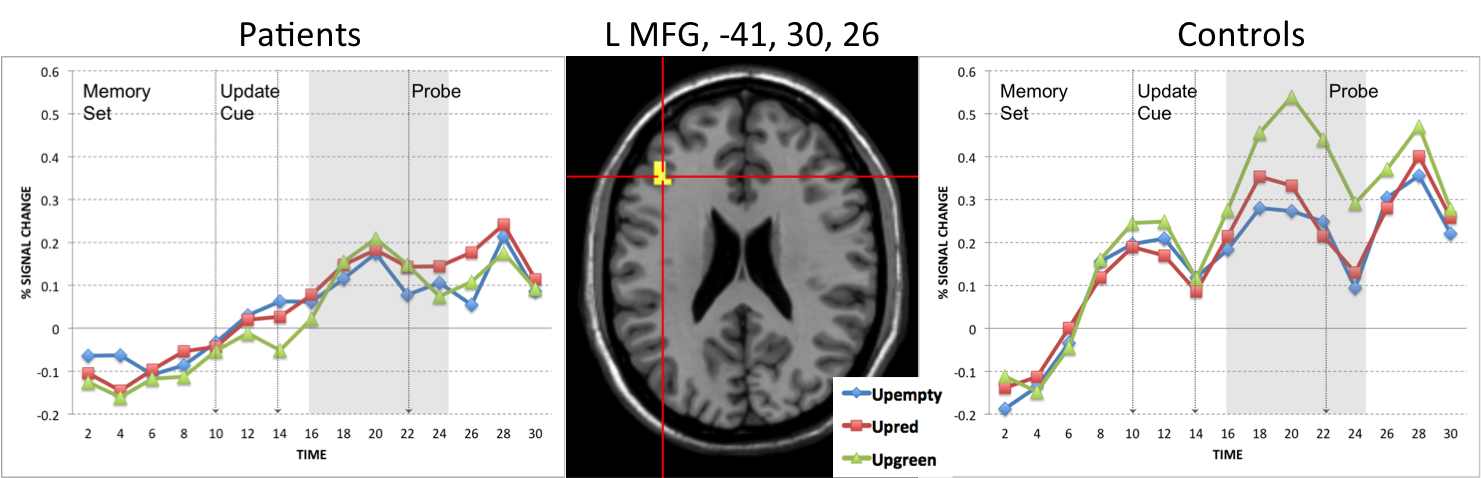


example, a lesion study of stroke patients found that lesions of the left lateral putamen and surrounding white matter resulted in deficits of distracter resistance (Baier et al., 2010), suggesting that the basal ganglia may play an important role in gating relevant information. Similarly, McNab et al. (McNab & Klingberg, 2008) found that the globus pallidus demonstrated selective associations with filtering unnecessary storage activity, suggesting that increases of globus pallidus activity may serve to filter our distracters from entering working memory storage. Other studies have shown that the dorsal striatum is involved in selective updating (Murty et al., 2011; Roth et al., 2006) and working memory manipulation (Lewis, Dove, Robbins, Barker, & Owen, 2004) when compared with other cognitive control processes. A study of dopamine depletion in the caudate of marmoset monkeys demonstrated the importance of dopamine when executing these cognitive processes, as the monkeys demonstrated selective deficits of a delayed response task but preserved attentional set-shifting (Collins, Wilkinson, Everitt, Robbins, & Roberts, 2000), although dopamine depletion influencing updating may not be the only explanation of these results.

Using the regions from our previous study we examined whether healthy controls from our current data set demonstrated the same pattern of condition effects. We again found that healthy control participants demonstrated greater prefrontal and striatal activity during updating than simple maintenance conditions. We also found some evidence that prefrontal activity in these independently defined regions demonstrated some transient activity to distracter presentation, consistent with our previous findings, though not all of the prefrontal regions demonstrated this effect. We did not find a significant response to distracter presentation within any of the striatal regions. These findings are partially consistent with the findings from our previous study, and suggest that while both the cortex and striatum process updating demands, the striatum may selectively activate and the PFC, particularly the IFG, may demonstrate more general condition sensitivity. For one of the independently defined striatal regions (right lateral caudate) we found maintenance activity that was intermediate to interference control and update activity. This was unexpected, given that participants were not presented with any shapes during the maintenance condition and maintenance activity that was greater than interference control activity was not something we found during our previous study. Because we used maintenance trials as our comparison condition increased activity during this condition may explain why we did not find differences between maintenance and updating or interference control. This finding is discussed in more detail below.

### 5.1.2. Anatomical Mask of Basal Ganglia and Prefrontal Cortex

In this analysis we identified regions that demonstrated condition effects (instead of examining condition effects only within regions defined in the prior study) and found multiple regions within the left and right MFG demonstrated condition effects in response to the presentation of the update cue, as did one region in the left caudate. The pattern for regions within the MFG was that updating activity was significantly greater than maintenance, with no difference between interference control and maintenance. While interference control activity in this region was numerically higher than maintenance activity (Replication Figure 2A) following presentation of the update cue, the fact that we did not find a significant difference between these conditions is not consistent with our previous work. It may be the case, however, that we generally lacked the power reliably to detect this subtle difference between the transient cortical response to distracter presentation and activity during the maintenance condition, even though our control sample in this data set is larger than the previous data set. That is, we found the same relative patterns of activity that we did in our previous study but given the difference between interference control and maintenance activity is small although the difference exists the significance of this difference may vary across smaller samples. The region in the caudate also demonstrated greater updating than maintenance activity, but greater maintenance activity when compared with interference control activity. One explanation for this increase of activity during maintenance trails compared to interference trials within the striatum is that it may reflect a salient cue orientation signal (Redgrave, Gurney, & Reynolds, 2008), as discussed next.

We used maintenance as a comparison condition because, unlike updating and interference control conditions, no shapes are presented during the update cue period. We assumed that there should be little to no change in striatal activity during this period given that striatal neuron projecting to the direct pathway would not activate in the absence of stimulus presentation and striatal neurons projecting to the indirect pathway are tonically active, and would not demonstrate a change of activation. After reviewing plots of time courses during the three different update cue conditions we see that this is not the case, and for many regions there is in fact mild to moderate increases of brain activity during the update cue period of maintenance trials. While no shapes were presented during this time empty boxes that are either red or green in color are presented and this presentation may be sufficient to elicit an increase of striatal activity, particularly in the caudate given that the boxes are salient and behaviorally relevant. A study by Zink et al. (2003) examined in humans the possibility that the striatum may function to process salient events regardless of reward value, rather than coding rewards and reward-related stimuli. They examined striatal response to nonrewarding salient stimuli using fMRI while manipulating the behavioral relevance of stimuli by manipulating salience by manipulating the frequency of distracter occurrence (such that high frequency resulted in less salience) and the behavioral relevance of the distracter (distracters that required a response and those that did not). They found that activity in both the nucleus accumbens and caudate increased in response to high salience nonrewarding stimuli, but activity in the caudate only did so when the stimuli was behaviorally relevant. Thus, increased caudate activity we observed during maintenance may reflect the salient, behaviorally relevant, properties of the green and red boxes presented during maintenance trials. Further, increased maintenance activity compared with interference control activity may also reflect the increased salience of “no-shape” maintenance trials when compared with “ignore-shape” distracter trials, given that the majority of trials used in the task presented either one or two shapes and thus maintenance trials were less frequent.

## 5.2. Condition Discussion

We examined brain activity during updating, distracter presentation, and simple maintenance in both our independently defined ROIs and in an anatomical mask of the prefrontal cortex and the striatum. Given that patients with schizophrenia may have dysregulated striatal activity and that activity in the striatum may be associated with information gating, we predicted that patients would demonstrated an attenuated striatal response in response to updating demands and an attenuated response to task conditions cortically. We first examined diagnostic differences at the condition level to identify broad difference in responses to updating, distracter presentation, and simple maintenance in regions that were defined using a previous data set of healthy controls. Three regions demonstrated significant interactions of condition and diagnosis – two in the dorsolateral prefrontal cortex (DLPFC) and one region in the caudate. Patients demonstrated greater DLPFC activity for updating compared to maintenance activity and even significantly greater DLPFC activity during interference control when compared with maintenance. Within the caudate there were no differences between conditions for patients and a plot of the time course revealed poor separation between conditions following the update cue. Controls also demonstrated significantly greater update and interference control DLPFC activity when compared with maintenance and no difference between conditions in the caudate, however a plot of the time course for controls revealed that there was separation between conditions but maintenance activity in the caudate that was intermediate to update and interference control activity. We did find that update and interference control activity for controls in the caudate significantly differed when we compared them directly. When comparing neural response to task demands between diagnostic groups we found that updating activity within the IFG and MFG for controls was significantly greater than updating activity within these regions for patients, but that there was no difference between groups when examining interference control activity within these regions and no differences between groups when examining caudate activity. So, while updating activity in the DLPFC for patients was significantly greater than comparison conditions, updating activity was still significantly lower than for controls. In the anatomical mask analysis, we found only one region in the MFG (BA 9) that demonstrated an interaction of condition and diagnosis. In this region, controls demonstrated significantly greater updating activity when compared with maintenance (an numerically greater interference control activity compared with maintenance), whereas patients did not demonstrate significant differences between task conditions. When comparing the neural response to task demands between diagnosis groups we found no differences, although control activity to updating and interference control was numerically greater.

These results provide some support for the hypothesis that patients would demonstrate dysregulated striatal activity during cognitive control demands, as evidenced by poor discrimination during 3 different task conditions. Interestingly, this was true when examining correct trials and this lack of discrimination was truer for the striatum than it was for regions within the DLPFC when examining our independently defined regions (Replication Table 3), although activity for patients in one region of the DLPFC (MFG; -41, 30, 26; Replication Figure 3) also demonstrated poor discrimination between conditions. Importantly, however, we also saw evidence for altered activity in the DLPFC. Of the DLPFC regions that demonstrated an interaction of diagnosis by condition we also observed that the response to updating demands for patients within a region was numerically lower than controls. It is interesting that the regions demonstrating tasks effects within our anatomical masks correspond to segments of the striatum striatal that have anatomical and functional connectivity with the DLPFC. For example, Draganski et al. (2008) examined cortico-striatal connectivity using probabilistic tractography and a novel method of creating voxel-based connectivity profiles to represent projections from a source to multiple target regions, called voxel connectivity profiles, on magnetic resonance diffusion imaging data of 30 healthy subjects. The aim of the study was to compare basal ganglia and thalamic connectivity of humans with anatomical patterns demonstrated in nonhuman primates, and to provide evidence of pathways between spatially segregated regions of the basal ganglia/thalamus and cortical regions. Amongst other findings, they found that rostral and caudal regions within the caudate and putamen demonstrated strong connectivity with the DLPFC and orbital frontal cortex (OFC). These findings were supported by Barnes et al. (2010), who used a combination of resting state functional connectivity MRI and graph theoretic analyses to parcellate subcortical structures of individual subjects and found that the locations of significant cortical-basal ganglia functional connectivity was consistent with connectivity of basal ganglia segments described above. While there is good evidence for both anatomical and functional connectivity between segmented cortico-striatal loops, the nature of the relationship during cognitive control has yet to be fully elucidated. A critical question for future research is to what degree does altered activity in the striatum and the DLPFC reflect an abnormal functional loop, and whether some of the variance in DLPFC disruption in schizophrenia might actually reflect dysregulated striatal function.

One way that cortico-striatal loops may impact cognitive control is through information gating, which may be accomplished through dense dopaminergic innervation of the striatum that transiently strengthen inputs to the frontal cortex, and by extending models of disinhibitory gating from the motor literature. As described in the introduction section, this gating mechanism has been described computationally by Frank et al. (2001) and, more recently, by Hazy et al. (Hazy et al., 2007). In this model, dopamine based reinforcement-learning provides appropriate learning signals that train direct pathway medium spiny neurons (MSNs) in the dorsal striatum when to fire, inhibiting the substantia nigra, which then releases the thalamus from tonic inhibition. Thalamic disinhibition enables, but does not cause, excitation of a segregated cortico-striatal loop and thus an information update, the same way that disinhibition via the basal ganglia sets a pattern of motor readiness in premotor networks rather than generating a command for muscular contraction (Chevalier & Deniau, 1990). Striatal spiny neurons in the indirect pathway are in competition with neurons in the direct pathway as they promote greater inhibition of thalamic neurons. In the prefrontal cortex, robust maintenance occurs through a combination of recurrent excitatory connectivity and bistability, which is toggled to and from a maintenance state via input from the basal ganglia. Hazy et al. (2007) also suggests that actively maintained representations in the prefrontal cortex may demonstrate top-down biasing of processing in relevant brain areas (e.g. posterior cortex, hippocampus, and basal ganglia), which may occur only when output-generating laminae within frontal cortical columns reach a threshold via basal ganglia-thalamic input signals (Hazy et al., 2007). Similarly, others suggests that dopamine and basal ganglia output may function to stabilize the information gate during distraction by enhancing task relevant memories in the cortex (Gruber et al., 2006) or that output from the basal ganglia gradually trains or builds up representations in the prefrontal cortex, and that without this input cortical representations are not as robust or distinct (Miller, 2013).

Based on the relationship between the prefrontal cortex and the basal ganglia described in the models discussed above, certain predictions could be made about how these regions will behave during specific task conditions. For example, during information updating one can expect that MSNs in the direct pathway will activate more strongly than MSNs in the indirect pathway, resulting in inhibition of the substantia nigra, disinhibition of the thalamus, and activation of the prefrontal cortex region within that cortico-striatal loop. During interference control, however, without appropriate dopaminergic input, MSNs in the indirect pathway will continue their tonic inhibition of the substantia nigra, which leads to inhibition of the thalamus and cortical regions within that segregated loop. The prefrontal cortex will activate in response to a distracter, but without basal ganglia-thalamic input signals this activation will not reach threshold. Thus, if dopamine signaling in the striatum were disrupted, as is the case with psychosis, one may expect that striatal output would be affected, perhaps though increased competition between direct and indirect MSNs resulting in weaker activation in response to updating demands, and with weaker basal ganglia-thalamic output to the cortex the cortical threshold would be more difficult to meet. Our finding that within the striatum patients demonstrated poor discrimination between conditions and appear to have an attenuated cortical response to updating demands are consistent with these predictions.

The models also imply that increases of brain activity in dorsal striatal and prefrontal regions should be associated with an update occurring, regardless of whether the update should have happened, because the “gate” opens anytime information is admitted to working memory stores. Further, a failure to update should be associated with decreased striatal and prefrontal activity because the “gate” failed to open. If a participant were to inappropriately update a distracter, for example, we would expect to see similar patterns of prefrontal and striatal activity that we would see during an appropriate update. We explored this idea in our analysis of trial type accuracy.

**Replication References**

Abi-Dargham, A. (2004). Do we still believe in the dopamine hypothesis? New data bring new evidence. *The International Journal of Neuropsychopharmacology / Official Scientific Journal of the Collegium Internationale Neuropsychopharmacologicum (CINP)*, *7 Suppl 1*(5), S1–5. doi:10.1017/S1461145704004110

Abi-Dargham, A., Mawlawi, O., Lombardo, I., Gil, R., Martinez, D., Huang, Y., et al. (2002). Prefrontal dopamine D1 receptors and working memory in schizophrenia. *The Journal of Neuroscience : the Official Journal of the Society for Neuroscience*, *22*(9), 3708–3719. doi:20026302

Andreasen, N. C. (1983). *Scale for the assessment of negative symptoms*. Iowa City: University of Iowa.

Andreasen, N. C. (1984). *Scale for the Assessment of Positive Symptons:(SAPS)*. Iowa City: University of Iowa.

Baier, B., Karnath, H.-O., Dieterich, M., Birklein, F., Heinze, C., & Muller, N. (2010). Keeping Memory Clear and Stable--The Contribution of Human Basal Ganglia and Prefrontal Cortex to Working Memory. *The Journal of Neuroscience : the Official Journal of the Society for Neuroscience*, *30*(29), 9788–9792.

Barch, D. M., & Ceaser, A. (2012). Cognition in schizophrenia: core psychological and neural mechanisms. *Trends in Cognitive Sciences*, *16*(1), 27–34. doi:10.1016/j.tics.2011.11.015

Barnes, K. A., Cohen, A. L., Power, J. D., Nelson, S. M., Dosenbach, Y. B. L., Miezin, F. M., et al. (2010). Identifying Basal Ganglia divisions in individuals using resting-state functional connectivity MRI. *Frontiers in Systems Neuroscience*, *4*, 18. doi:10.3389/fnsys.2010.00018

Berk, M., Berk, L., Dodd, S., Fitzgerald, P. B., de Castella, A. R., Filia, S., et al. (2013). The sick role, illness cognitions and outcomes in bipolar disorder. *Journal of Affective Disorders*, *146*(1), 146–149. doi:10.1016/j.jad.2012.07.003

Berridge, K. C. (2007). The debate over dopamine's role in reward: the case for incentive salience. *Psychopharmacology*, *191*(3), 391–431. doi:10.1007/s00213-006-0578-x

Berridge, K. C., & Robinson, T. E. (1998). What is the role of dopamine in reward: hedonic impact, reward learning, or incentive salience? *Brain Research Brain Research Reviews*, *28*(3), 309–369.

Braver, T. S., & Cohen, J. (2000). On the control of control: The role of dopamine in regulating prefrontal function and working memory. *Control of Cognitive Processes: Attention and Performance XVIII*, 713–737.

Braver, T. S., Barch, D. M., & Cohen, J. D. (1999). Cognition and control in schizophrenia: a computational model of dopamine and prefrontal function. *Biological Psychiatry*, *46*(3), 312–328.

Braver, T. S., Cohen, J. D., Nystrom, L. E., Jonides, J., Smith, E. E., & Noll, D. C. (1997). A parametric study of prefrontal cortex involvement in human working memory. *NeuroImage*, *5*(1), 49–62. doi:10.1006/nimg.1996.0247

Breier, A., Schreiber, J. L., Dyer, J., & Pickar, D. (1991). National Institute of Mental Health longitudinal study of chronic schizophrenia. Prognosis and predictors of outcome. *Archives of General Psychiatry*, *48*(3), 239–246.

Buckner, R. L., Head, D., Parker, J., Fotenos, A. F., Marcus, D., Morris, J. C., & Snyder, A. Z. (2004). A unified approach for morphometric and functional data analysis in young, old, and demented adults using automated atlas-based head size normalization: reliability and validation against manual measurement of total intracranial volume. *NeuroImage*, *23*(2), 724–738.

Chapman, L. J., Edell, W. S., & Chapman, J. P. (1980). cic. *Schizophrenia Bulletin*, *6*(4), 639–653.

Chevalier, G., & Deniau, J. M. (1990). Disinhibition as a basic process in the expression of striatal functions. *Trends in Neurosciences VL -*, *13*(7), 277–280.

Cicero, D. C., Kerns, J. G., & McCarthy, D. M. (2010). The Aberrant Salience Inventory: a new measure of psychosis proneness. *Psychological Assessment*, *22*(3), 688–701. doi:10.1037/a0019913

Cohen, J. D., Braver, T. S., & O'Reilly, R. C. (1996). A computational approach to prefrontal cortex, cognitive control and schizophrenia: recent developments and current challenges. *Philosophical Transactions of the Royal Society of London Series B, Biological Sciences*, *351*(1346), 1515–1527. doi:10.1098/rstb.1996.0138

Collette, F., Van der Linden, M., Laureys, S., Arigoni, F., Delfiore, G., Degueldre, C., et al. (2007). Mapping the updating process: common and specific brain activations across different versions of the running span task. *Cortex; a Journal Devoted to the Study of the Nervous System and Behavior*, *43*(1), 146–158.

Collin, C. A., & McMullen, P. A. (2002). Using Matlab to generate families of similar Attneave shapes. *Behavior Research Methods, Instruments, & Computers : a Journal of the Psychonomic Society, Inc*, *34*(1), 55–68.

Collins, P., Wilkinson, L. S., Everitt, B. J., Robbins, T. W., & Roberts, A. C. (2000). The effect of dopamine depletion from the caudate nucleus of the common marmoset (Callithrix jacchus) on tests of prefrontal cognitive function. *Behavioral Neuroscience*, *114*(1), 3–17.

Cools, R. (2008). Role of dopamine in the motivational and cognitive control of behavior. *The Neuroscientist : a Review Journal Bringing Neurobiology, Neurology and Psychiatry*, *14*(4), 381–395. doi:10.1177/1073858408317009

Corlett, P. R., & Fletcher, P. C. (2012). The neurobiology of schizotypy: fronto-striatal prediction error signal correlates with delusion-like beliefs in healthy people. *Neuropsychologia*, *50*(14), 3612–3620. doi:10.1016/j.neuropsychologia.2012.09.045

Davis, K., Kahn, R., Ko, G., & Davidson, M. (1991). Dopamine in schizophrenia: a review and reconceptualization. *The American Journal of Psychiatry*.

Demjaha, A., Egerton, A., Murray, R. M., Kapur, S., Howes, O. D., Stone, J. M., & McGuire, P. K. (2014). Antipsychotic treatment resistance in schizophrenia associated with elevated glutamate levels but normal dopamine function. *Biological Psychiatry*, *75*(5), e11–3. doi:10.1016/j.biopsych.2013.06.011

Demjaha, A., Murray, R. M., McGuire, P. K., Kapur, S., & Howes, O. D. (2012). Dopamine synthesis capacity in patients with treatment-resistant schizophrenia. *The American Journal of Psychiatry*, *169*(11), 1203–1210. doi:10.1176/appi.ajp.2012.12010144

Dickinson, D., Ragland, J. D., Gold, J. M., & Gur, R. C. (2008). General and specific cognitive deficits in schizophrenia: Goliath defeats David? *Biological Psychiatry*, *64*(9), 823–827. doi:10.1016/j.biopsych.2008.04.005

Dickinson, D., Ramsey, M. E., & Gold, J. M. (2007). Overlooking the obvious: a meta-analytic comparison of digit symbol coding tasks and other cognitive measures in schizophrenia. *Archives of General Psychiatry*, *64*(5), 532–542. doi:10.1001/archpsyc.64.5.532

Draganski, B., Kherif, F., Klöppel, S., Cook, P. A., Alexander, D. C., Parker, G. J. M., et al. (2008). Evidence for segregated and integrative connectivity patterns in the human Basal Ganglia. *The Journal of Neuroscience : the Official Journal of the Society for Neuroscience*, *28*(28), 7143–7152. doi:10.1523/JNEUROSCI.1486-08.2008

Egerton, A., Chaddock, C. A., Winton-Brown, T. T., Bloomfield, M. A. P., Bhattacharyya, S., Allen, P., et al. (2013). Presynaptic striatal dopamine dysfunction in people at ultra-high risk for psychosis: findings in a second cohort. *Biological Psychiatry*, *74*(2), 106–112. doi:10.1016/j.biopsych.2012.11.017

Esslinger, C., Englisch, S., Inta, D., Rausch, F., Schirmbeck, F., Mier, D., et al. (2012). Ventral striatal activation during attribution of stimulus saliency and reward anticipation is correlated in unmedicated first episode schizophrenia patients. *Schizophrenia Research*, *140*(1-3), 114–121. doi:10.1016/j.schres.2012.06.025

Ettinger, U., Corr, P. J., Mofidi, A., Williams, S. C. R., & Kumari, V. (2013). Dopaminergic basis of the psychosis-prone personality investigated with functional magnetic resonance imaging of procedural learning. *Frontiers in Human Neuroscience*, *7*, 130. doi:10.3389/fnhum.2013.00130

First, M. B., Spitzer, R. L., Gibbon, M., & Williams, J. B. (2002). *Structured clinical interview for DSM-IV-TR axis I disorders, research version, patient edition*. New York: New York: Biometrics Research, New York State Psychiatric Institute.

Frank, M. J., Loughry, B., & O'Reilly, R. C. (2001). Interactions between frontal cortex and basal ganglia in working memory: a computational model. *Cognitive, Affective & Behavioral Neuroscience*, *1*(2), 137–160.

Goldman-Rakic, P. S. (1995). Cellular basis of working memory. *Neuron*, *14*(3), 477–485.

Goldman-Rakic, P. S., Castner, S. A., Svensson, T. H., Siever, L. J., & Williams, G. V. (2004). Targeting the dopamine D1 receptor in schizophrenia: insights for cognitive dysfunction. *Psychopharmacology*, *174*(1), 3–16. doi:10.1007/s00213-004-1793-y

Gruber, A. J., Dayan, P., Gutkin, B. S., & Solla, S. A. (2006). Dopamine modulation in the basal ganglia locks the gate to working memory. *Journal of Computational Neuroscience*, *20*(2), 153–166. doi:10.1007/s10827-005-5705-x

Hazy, T. E., Frank, M. J., & O'Reilly, R. C. (2006). Banishing the homunculus: making working memory work. *Neuroscience*, *139*(1), 105–118. doi:10.1016/j.neuroscience.2005.04.067

Hazy, T. E., Frank, M. J., & O'Reilly, R. C. (2007). Towards an executive without a homunculus: computational models of the prefrontal cortex/basal ganglia system. *Philosophical Transactions of the Royal Society of London Series B, Biological Sciences*, *362*(1485), 1601–1613. doi:10.1098/rstb.2007.2055

Howes, O. D., & Kapur, S. (2009). The dopamine hypothesis of schizophrenia: version III--the final common pathway. *Schizophrenia Bulletin*, *35*(3), 549–562. doi:10.1093/schbul/sbp006

Howes, O. D., Bose, S. K., Turkheimer, F., Valli, I., Egerton, A., Valmaggia, L. R., et al. (2011). Dopamine synthesis capacity before onset of psychosis: a prospective [18F]-DOPA PET imaging study. *The American Journal of Psychiatry*, *168*(12), 1311–1317. doi:10.1176/appi.ajp.2011.11010160

Howes, O. D., Kambeitz, J., Kim, E., Stahl, D., Slifstein, M., Abi-Dargham, A., & Kapur, S. (2012). The nature of dopamine dysfunction in schizophrenia and what this means for treatment. *Archives of General Psychiatry*, *69*(8), 776–786. doi:10.1001/archgenpsychiatry.2012.169

Howes, O. D., Montgomery, A. J., Asselin, M.-C., Murray, R. M., Valli, I., Tabraham, P., et al. (2009). Elevated striatal dopamine function linked to prodromal signs of schizophrenia. *Archives of General Psychiatry*, *66*(1), 13–20. doi:10.1001/archgenpsychiatry.2008.514

Jensen, J., Willeit, M., Zipursky, R. B., Savina, I., Smith, A. J., Menon, M., et al. (2008). The formation of abnormal associations in schizophrenia: neural and behavioral evidence. *Neuropsychopharmacology : Official Publication of the American College of Neuropsychopharmacology*, *33*(3), 473–479. doi:10.1038/sj.npp.1301437

Johnson-Selfridge, M., & Zalewski, C. (2001). Moderator variables of executive functioning in schizophrenia: meta-analytic findings. *Schizophrenia Bulletin*, *27*(2), 305–316.

Kapur, S. (2003). Psychosis as a state of aberrant salience: a framework linking biology, phenomenology, and pharmacology in schizophrenia. *The American Journal of Psychiatry*, *160*(1), 13–23.

Karlsson, P., Farde, L., Halldin, C., & Sedvall, G. (2002). PET study of D(1) dopamine receptor binding in neuroleptic-naive patients with schizophrenia. *The American Journal of Psychiatry*, *159*(5), 761–767.

Kegeles, L. S., Abi-Dargham, A., Frankle, W. G., Gil, R., Cooper, T. B., Slifstein, M., et al. (2010). Increased synaptic dopamine function in associative regions of the striatum in schizophrenia. *Archives of General Psychiatry*, *67*(3), 231–239. doi:10.1001/archgenpsychiatry.2010.10

Kwapil, T. R. (1998). Social anhedonia as a predictor of the development of schizophrenia-spectrum disorders. *Journal of Abnormal Psychology*, *107*(4), 558.

Lewis, S. J. G., Dove, A., Robbins, T. W., Barker, R. A., & Owen, A. M. (2004). Striatal contributions to working memory: a functional magnetic resonance imaging study in humans. *The European Journal of Neuroscience*, *19*(3), 755–760.

Linden, D. E. J. (2007). The Working Memory Networks of the Human Brain. *The Neuroscientist : a Review Journal Bringing Neurobiology, Neurology and Psychiatry*, *13*(3), 257–267. doi:10.1177/1073858406298480

Linscott, R. J., & van Os, J. (2010). Systematic reviews of categorical versus continuum models in psychosis: evidence for discontinuous subpopulations underlying a psychometric continuum. Implications for DSM-V, DSM-VI, and DSM-VII. *Annual Review of Clinical Psychology*, *6*(1), 391–419. doi:10.1146/annurev.clinpsy.032408.153506

Martinez-Aran, A., Penadés, R., Vieta, E., Colom, F., Reinares, M., Benabarre, A., et al. (2002). Executive function in patients with remitted bipolar disorder and schizophrenia and its relationship with functional outcome. *Psychotherapy and Psychosomatics*, *71*(1), 39–46.

Martinez-Aran, A., Vieta, E., Torrent, C., Sanchez-Moreno, J., Goikolea, J. M., Salamero, M., et al. (2007). Functional outcome in bipolar disorder: the role of clinical and cognitive factors. *Bipolar Disorders*, *9*(1-2), 103–113. doi:10.1111/j.1399-5618.2007.00327.x

McAvoy, M., Ollinger, J., & Buckner, R. (2001). Cluster size thresholds for assessment of significant activation in fMRI. *NeuroImage*, *13*(6, Supplement), 198 EP–. doi:doi: 10.1016/S1053-8119(01)91541-1

McClure, R., & Lieberman, J. (2003). Neurodevelopmental and neurodegenerative hypotheses of schizophrenia: a review and critique. *Current Opinion in Psychiatry*, *16*, S15.

McNab, F., & Klingberg, T. (2008). Prefrontal cortex and basal ganglia control access to working memory. *Nature Neuroscience*, *11*(1), 103–107. doi:10.1038/nn2024

Meng, X.-L., Rosenthal, R., & Rubin, D. B. (1992). Comparing correlated correlation coefficients. *Psychological Bulletin*, *111*(1), 172–175. doi:10.1037/0033-2909.111.1.172

Middleton, F. A., & Strick, P. L. (1994). Anatomical evidence for cerebellar and basal ganglia involvement in higher cognitive function. *Science*, *266*(5184), 458–461.

Miller, E. K. (2013). The “working” of working memory. *Dialogues in Clinical Neuroscience*, *15*(4), 411–418.

Minzenberg, M. J., Laird, A. R., Thelen, S., Carter, C. S., & Glahn, D. C. (2009). Meta-analysis of 41 functional neuroimaging studies of executive function in schizophrenia. *Archives of General Psychiatry*, *66*(8), 811–822. doi:10.1001/archgenpsychiatry.2009.91

Miyake, A., Friedman, N. P., Emerson, M. J., Witzki, A. H., Howerter, A., & Wager, T. D. (2000). The unity and diversity of executive functions and their contributions to complex “Frontal Lobe” tasks: a latent variable analysis. *Cognitive Psychology*, *41*(1), 49–100. doi:10.1006/cogp.1999.0734

Moore, H., Jentsch, J. D., Ghajarnia, M., Geyer, M. A., & Grace, A. A. (2006). A neurobehavioral systems analysis of adult rats exposed to methylazoxymethanol acetate on E17: implications for the neuropathology of schizophrenia. *Biological Psychiatry*, *60*(3), 253–264. doi:10.1016/j.biopsych.2006.01.003

Morris, R., Griffiths, O., Le Pelley, M. E., & Weickert, T. W. (2012). Attention to Irrelevant Cues Is Related to Positive Symptoms in Schizophrenia. *Schizophrenia Bulletin*. doi:10.1093/schbul/sbr192

Murty, V. P., Sambataro, F., Radulescu, E., Altamura, M., Iudicello, J., Zoltick, B., et al. (2011). Selective updating of working memory content modulates meso-cortico-striatal activity. *NeuroImage*, *57*(3), 1264–1272. doi:10.1016/j.neuroimage.2011.05.006

Norman, D., & Shallice, T. (2000). Attention io Action: Willed and Auiomatic Control of Behavior. *Cognitive Neuroscience: a Reader*, *376*.

Nuechterlein, K. H., Subotnik, K. L., Green, M. F., Ventura, J., Asarnow, R. F., Gitlin, M. J., et al. (2011). Neurocognitive predictors of work outcome in recent-onset schizophrenia. *Schizophrenia Bulletin*, *37 Suppl 2*, S33–40. doi:10.1093/schbul/sbr084

O'Doherty, J. P., Dayan, P., Friston, K., Critchley, H., & Dolan, R. J. (2003). Temporal difference models and reward-related learning in the human brain. *Neuron*, *38*(2), 329–337.

Oberauer, K. (2009). *Design for a Working Memory*. *Psychology of Learning and Motivation* (Vol. 51, pp. 45–100). doi:DOI: 10.1016/S0079-7421(09)51002-X

Ojemann, J. G., Akbudak, E., Snyder, A. Z., McKinstry, R. C., Raichle, M. E., & Conturo, T. E. (1997). Anatomic localization and quantitative analysis of gradient refocused echo-planar fMRI susceptibility artifacts. *NeuroImage*, *6*(3), 156–167.

Okubo, Y., Suhara, T., Suzuki, K., Kobayashi, K., Inoue, O., Terasaki, O., et al. (1997). Decreased prefrontal dopamine D1 receptors in schizophrenia revealed by PET. *Nature*, *385*(6617), 634–636. doi:10.1038/385634a0

Ollinger, J., Corbetta, M., & Shulman, G. (2001). Separating Processes within a Trial in Event-Related Functional MRI: II. Analysis. *NeuroImage*, *13*(1), 218–229. doi:doi: 10.1006/nimg.2000.0711

Palmiter, R. D. (2008). Dopamine signaling in the dorsal striatum is essential for motivated behaviors: lessons from dopamine-deficient mice. *Annals of the New York Academy of Sciences*, *1129*(1), 35–46. doi:10.1196/annals.1417.003

Parnas, J., Handest, P., Saebye, D., & Jansson, L. (2003). Anomalies of subjective experience in schizophrenia and psychotic bipolar illness. *Acta Psychiatrica Scandinavica*, *108*(2), 126–133.

Raine, A. (1991). The SPQ: a scale for the assessment of schizotypal personality based on DSM-III-R criteria. *Schizophrenia Bulletin*, *17*(4), 555.

Rajkowska, G., & Goldman-Rakic, P. S. (1995). Cytoarchitectonic definition of prefrontal areas in the normal human cortex: I. Remapping of areas 9 and 46 using quantitative criteria. *Cerebral Cortex (New York, NY : 1991)*, *5*(4), 307–322.

Randall, C., & Munakata, Y. (2000). *Computational Explorations in Cognitive Neuroscience: Understanding the Mind by Simulating the Brain*.

Redgrave, P., Gurney, K., & Reynolds, J. (2008). What is reinforced by phasic dopamine signals? *Brain Research Reviews*, *58*(2), 322–339. doi:10.1016/j.brainresrev.2007.10.007

Reichenberg, A., & Harvey, P. D. (2007). Neuropsychological impairments in schizophrenia: Integration of performance-based and brain imaging findings. *Psychological Bulletin*, *133*(5), 833–858. doi:10.1037/0033-2909.133.5.833

Resnick SM. (1992). MAtching for education in studies of schizophrenia. *Archives of General Psychiatry*, *49*(3), 246–246. doi:doi: 10.1001/archpsyc.1992.01820030078011

Roberts, G. (1992). The origins of delusion. *The British Journal of Psychiatry : the Journal of Mental Science*, *161*, 298–308.

Roiser, J. P., Howes, O. D., Chaddock, C. A., Joyce, E. M., & McGuire, P. (2013). Neural and behavioral correlates of aberrant salience in individuals at risk for psychosis. *Schizophrenia Bulletin*, *39*(6), 1328–1336. doi:10.1093/schbul/sbs147

Roiser, J. P., Stephan, K. E., Ouden, den, H. E. M., Barnes, T. R. E., Friston, K. J., & Joyce, E. M. (2009). Do patients with schizophrenia exhibit aberrant salience? *Psychological Medicine*, *39*(2), 199–209. doi:10.1017/S0033291708003863

Roiser, J. P., Stephan, K. E., Ouden, den, H. E. M., Friston, K. J., & Joyce, E. M. (2010). Adaptive and aberrant reward prediction signals in the human brain. *NeuroImage*, *50*(2), 657–664. doi:10.1016/j.neuroimage.2009.11.075

Rosell, A., & Giménez-Amaya, J. M. (1999). Anatomical re-evaluation of the corticostriatal projections to the caudate nucleus: a retrograde labeling study in the cat. *Neuroscience Research*, *34*(4), 257–269.

Roth, J. K., Serences, J. T., & Courtney, S. M. (2006). Neural system for controlling the contents of object working memory in humans. *Cerebral Cortex (New York, NY : 1991)*, *16*(11), 1595–1603. doi:10.1093/cercor/bhj096

Rougier, N. P., Noelle, D. C., Braver, T. S., Cohen, J. D., & O'Reilly, R. C. (2005). Prefrontal cortex and flexible cognitive control: rules without symbols. *Proceedings of the National Academy of Sciences of the United States of America*, *102*(20), 7338–7343. doi:10.1073/pnas.0502455102

Schmidt, K., & Roiser, J. P. (2009). Assessing the construct validity of aberrant salience. *Frontiers in Behavioral Neuroscience*, *3*, 58. doi:10.3389/neuro.08.058.2009

Sheehan, D. V., Lecrubier, Y., Sheehan, K. H., Amorim, P., Janavs, J., Weiller, E., et al. (1998). The Mini-International Neuropsychiatric Interview (MINI): the development and validation of a structured diagnostic psychiatric interview for DSM-IV and ICD-10. *Journal of Clinical Psychiatry*, *59*, 22–33.

Siegel, J. S., Power, J. D., Dubis, J. W., Vogel, A. C., Church, J. A., Schlaggar, B. L., & Petersen, S. E. (2013). Statistical improvements in functional magnetic resonance imaging analyses produced by censoring high-motion data points. *Human Brain Mapping*. doi:10.1002/hbm.22307

Smith, Y., Bevan, M. D., Shink, E., & Bolam, J. P. (1998). Microcircuitry of the direct and indirect pathways of the basal ganglia. *Neuroscience*, *86*(2), 353–387.

Talairach, J., & Tournoux, P. (1988). *Co-planar stereotactic atlas of the human brain, 1988*. New York: Theime Medical.

Tanaka, S. (2002). Dopamine controls fundamental cognitive operations of multi-target spatial working memory. *Neural Networks : the Official Journal of the International Neural Network Society*, *15*(4-6), 573–582.

Van Snellenberg, J. X., Torres, I. J., & Thornton, A. E. (2006). Functional neuroimaging of working memory in schizophrenia: task performance as a moderating variable. *Neuropsychology*, *20*(5), 497–510. doi:10.1037/0894-4105.20.5.497

Ventura, J., Hellemann, G. S., Thames, A. D., Koellner, V., & Nuechterlein, K. H. (2009). Symptoms as mediators of the relationship between neurocognition and functional outcome in schizophrenia: a meta-analysis. *Schizophrenia Research*, *113*(2-3), 189–199. doi:10.1016/j.schres.2009.03.035

Wang, L., Mamah, D., Harms, M. P., Karnik, M., Price, J. L., Gado, M. H., et al. (2008). Progressive deformation of deep brain nuclei and hippocampal-amygdala formation in schizophrenia. *Biological Psychiatry*, *64*(12), 1060–1068. doi:10.1016/j.biopsych.2008.08.007

Wechsler, D. (1997). *WAIS-III: Wechsler adult intelligence scale*. San Antonio, TX: Psychological Corporation.

Zink, C. F., Pagnoni, G., Martin, M. E., Dhamala, M., & Berns, G. S. (2003). Human striatal response to salient nonrewarding stimuli. *The Journal of Neuroscience : the Official Journal of the Society for Neuroscience*, *23*(22), 8092–8097.

**Replication Figure Captions**

Replication Figure 1: **Brain Activity of Healthy Controls and Patients Within Regions Defined in a Previous Data Set.** The figures above list the full trial time course of brain activity of healthy controls and patients from the current data set within regions defined in a previous data set. Green lines represent Upgreen activity, red lines represent Upred activity, and blue lines represent Upempty activity. “Memory Set” in the figure denotes the period during which the memory set items are presented. “Update Cue” in the figure and the two arrow lines represent the onset (10 seconds) and offset (13 seconds) of the update cue event. The gray box (16-24 seconds) represents the time frame used in our follow up update cue analyses (corresponding to frames 8-12), which is shifted from the offset of the update cue to account for hemodynamic lag. “Probe” in the figure and the arrow line at the 22 second time point indicate the onset of the probe. We plotted the time course of brain activity for patients and controls for two representative regions, left lateral inferior frontal gyrus (IFG) and left lateral putamen, that demonstrated significant effects of condition for healthy controls went on to significantly interact with diagnosis. The regions that time courses were taken from appear in the cross hairs of the brain figure. Controls are listed in the first column (Replication Figure 1A and 3C) and patients are listed in the second column (Replication Figure 1B and 3D). These regions were selected because they represent a region in the frontal cortex and the striatum that demonstrated condition effects for controls. For controls we observed significant differences between Upgreen and Upempty during frames 8-12 (16-24 seconds) for the IFG (-39, 4, 30) and putamen (-18, -3, 13). We also observed a significant difference between Upred and Upempty for the region in the IFG, but not the putamen region for controls. The region in the IFG went on to interact with diagnosis. Patients demonstrated a significant difference between Upgreen and Upempty during frames 8-12 (16-24 seconds) for the IFG (-39, 4, 30) and a trend towards difference between Upred and Upempty (*F*(1,21) = 3.06, *p* = 0.095). For putamen activity of patients, we observed no differences between either Upgreen and Upempty or Upred and Upempty.

Replication Figure 2: **Regions of Healthy Controls from the Current Data Set that Demonstrated Effects of Condition.** Time courses for representative regions from the frontal cortex and striatum derived from our current sample of controls that demonstrated condition effects following the update cue. Green lines represent Upgreen activity, red lines represent Upred activity, and blue lines represent Upempty activity. We found that both regions demonstrated significant differences between Upgreen and Upempty, but only the caudate demonstrated differences between Upred and Upempty. However, we found the unexpected pattern that Upempty activity was greater than Upred activity.

Replication Figure 3: **Frontal Region from the Current Data Set that Demonstrated A Diagnosis by Condition Interaction.** The time courses for a region in the middle frontal gyrus that demonstrated an interaction of diagnosis and condition during frames 8-12 (gray box in the figure). Green lines represent Upgreen activity, red lines represent Upred activity, and blue lines represent Upempty activity. Again, we found that control participants demonstrated significantly greater Upgreen versus Upempty activity in this region, and Upred activity was intermediate to Upgreen and Upred (the difference between Upred and Upempty, however, was not significant). Patients, however, did not demonstrate a difference between the three condition types.
